# The mid‐domain effect and habitat complexity applied to elevational gradients: Moss species richness in a temperate semihumid monsoon climate mountain of China

**DOI:** 10.1002/ece3.7576

**Published:** 2021-05-04

**Authors:** De Gao, Liqin Fu, Jiaxing Sun, Yan Li, Zhen Cao, Yongying Liu, Peng Xu, Jiancheng Zhao

**Affiliations:** ^1^ Department of Resources and Environmental Science Hebei Normal University Shijiazhuang China; ^2^ Hebei Key Laboratory of Environmental Change and Ecological Construction Shijiazhuang China; ^3^ Hebei Technology Innovation Center for Remote Sensing Identification of Environmental Change Shijiazhuang China; ^4^ Department of Life Science Hebei Normal University Shijiazhuang China; ^5^ Department of Chemical and Environmental Engineering Hebei College of Industry and Technology Shijiazhuang China; ^6^ Department of Biology Jiaozuo Normal College Jiaozuo China; ^7^ Department of Mathematics and Statistics Eastern Michigan University Ypsilanti MI USA

**Keywords:** elevational gradient, habitat complexity, heterogeneity, mid‐domain effect, moss species

## Abstract

The utility of elevational gradients as tools to test either ecological hypotheses and delineate elevation‐associated environmental factors that explain the species diversity patterns is critical for moss species conservation. We examined the elevational patterns of species richness and evaluated the effects of spatial and environmental factors on moss species predicted a priori by alternative hypotheses, including mid‐domain effect (MDE), habitat complexity, energy, and environment proposed to explain the variation of diversity. Last, we assessed the contribution of elevation toward explaining the heterogeneity among sampling sites. We observed the hump‐shaped distribution pattern of species richness along elevational gradient. The MDE and the habitat complexity hypothesis were supported with MDE being the primary driver for richness patterns, whereas little support was found for the energy and the environmental factors.

## Graphical abstract

We observed the hump‐shaped distribution pattern of species richness along elevational gradient. The mid‐domain effect (MDE) and the habitat complexity hypothesis were supported with MDE being the primary driver for richness patterns, whereas little support was found for the energy and the environment. Moss species richness pattern in the mountain is driven by ecological and evolutionary effects, whereas evolutionary factors predominately shape the large heterogeneity through dispersal, extinction, and speciation processes.

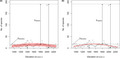

## 1 INTRODUCTION

Understanding biogeographic variation in species diversity patterns is important for conservation of biological diversity (Socolar et al., 2016; Vetaas & Grytnes, 2002). Elevational patterns of species richness, in recent decades, have received much attention in ecological and biogeographic studies (Brown, 2001; McCain, 2004; Stevens et al., 2019), given the advantages of elevational gradients, such as global ubiquity and smaller spatial scale (Rahbek, 2005; Wu et al., 2013). Moreover, many of the world's biodiversity hotspots are associated with montane regions, resulting in a crucial importance for biogeography, biodiversity, and conservation research to understand the underlying mechanisms of montane diversity (Fjeldså et al., 2012). For these reasons, a growing body of research is focusing on the utility of elevational gradients as a tool for testing ecological hypotheses and uncovering the mechanisms and constraints that shape both patterns of biodiversity and the functioning of ecosystems (Colwell & Lees, 2000; Lomolino, 2001; McCain, 2004, 2009; Phillips et al., 2019; Rahbek, 1995, 2005; Wu et al., 2013) in various taxa, such as fungi (Geml et al., 2017), plants (Gong et al., 2019; Wang et al., 2007), insects (Brehm et al., 2007), small mammals (Wu et al., 2013), birds (Kattan & Franco, 2004; Wu et al., 2014), and reptiles (McCain, 2010).

Numerous hypotheses have been proposed to explain species richness patterns along elevational gradients, but no one is consistently supported with empirical data (Nascimbene & Marini, 2015; Raabe et al., 2010; Spitale, 2016). Specifically, the mid‐domain effect (MDE) indicates that if species' ranges are distributed randomly within a bounded domain, more ranges will overlap in the middle of the domain than at the edges which will produce a hump‐shaped pattern of species richness (Colwell & Hurtt, 1994; Colwell & Lees, 2000). The energy hypothesis proposes that higher ambient energy and productivity often results in higher species diversity (Hawkins et al., 2003). The environment hypothesis proposes that species richness patterns are generated by the climatic factors such as rainfall, temperature, and water availability (Heaney, 2001; McCain, 2007; Sánchez‐Cordero, 2001). Moreover, habitat complexity has also been regarded as a potential driver of species richness (Brown, 2001; Wu et al., 2013).

Although the mechanism of geographic variation in species richness is important and has been explored by ecologists for decades, there are still limitations of elevational richness patterns. Species are generally not homogeneously distributed along elevational gradients, and the heterogeneity in biodiversity within (*α*) and among (*β*) sampling sites cannot be revealed by the elevational richness pattern. As envisioned by the combination of additive diversity partitioning and species–area relationship, β‐diversity among sampling sites may partly be explained by a factor gradient (Gao & Perry, 2016; Golodets et al., 2011; Zajac et al., 2013); thereby, we suggest elevational richness pattern alone cannot quantify how much of the total β‐diversity is due to elevation (*β*_elevation_) and how much is due to other factors (*β*_replace_). Moreover, the comparison of the diversity within (*α*) and among (*β*) sampling sites and the contributions made by elevation (*β*_elevation_) and other factors (*β*_replace_) are important for strategic conservation planning. A low α‐diversity with a high β‐diversity suggests that species assemblages are heterogeneous and species are often specific to individual sampling sites, while a high α‐diversity with a low β‐diversity indicates that species assemblages are homogenous and species within each sampling site are a subsample of the same species pool (Francisco‐Ramos & Arias‐González, 2013). A high *β*_elevation_ with a low *β*_replace_ indicates that species richness varies in a more predictable manner determined by factors that have a strong association with elevation, while a low *β*_elevation_ with a high *β*_replace_ suggests that factors such as speciation, dispersal, and extinction have a greater role in influencing patterns of β‐diversity (Rahbek, Borregaard, Antonelli, et al., 2019).

The majority of elevational richness pattern studies on flora have focused on vascular plants (e.g., Bhattarai & Vetaas, 2003; Kessler et al., 2011; Kitayama, 1992; Liberman et al., 1996; Nervo et al., 2019), although the success of land plants is apparent in the diversification of the nonvascular mosses (Bryophyta) with over 12,700 species worldwide (Crosby et al., 1999; Laenen et al., 2014). In contrast to many vascular plants, mosses are dispersed by means of small spores and establish new populations in distant localities. They colonize almost all terrestrial habitats, exhibit less frequent speciation, and have a long evolutionary history. Because of these unique features, our aims were to (a) depict the species richness pattern of mosses along elevational gradient, (b) evaluate the importance of four ecological hypotheses in predicting variation of moss species diversity along elevational gradient, and (c) examine how much contribution that elevation made toward explaining the among‐sampling heterogeneity.

## 2 MATERIALS AND METHODS

### 2.1 Study area

This study was conducted on the Mt Tuofeng (maximum elevation 2,282 m a.s.l.). It sits within the Tuoliang National Reserve, in the middle of the Taihang Mountains in central west Hebei Province, China (38°33′–38°45′N, 113°41′–113°53′E). The study area is in the transition between warm and cold temperate zone and generally has a semihumid semiarid continental monsoon mountain climate with four distinct seasons, abundant sunshine, large temperature difference between day and night, moderate rainfall, and an annual average temperature (AT) of 8.0°C.

### 2.2 Sampling and species identification

The present study was conducted along the elevational gradient of the Mt Tuofeng between 923 and 2,282 m a.s.l. during July–September 2018 within Tuoliang National Reserve. We randomly selected 73 sampling sites (10 × 10 m) from three transects along the elevational gradient to cover all types of vegetation with an equal elevational distance (c. 19 m) and over 100 m apart between each other. In each site, we collected all moss species from the ground to two meters above the ground. Moss specimens were taken back to the laboratory of Hebei Normal University where all species were identified from October 2018 to May 2019. Finally, the outcome of species occurrence for each sampling site is summarized in Table S1.

### 2.3 Ecological variables: MDE, habitat, and climate

We utilized the MDE model in RangeModel ver. 5 (Colwell, 2006) to test the MDE. We employed the discrete domain analysis for the sampling sites, which in our study were discrete and evenly spaced. Species richness data for each sampling site were compared with null model predictions using a Monte Carlo simulation of species richness curves to evaluate the explanatory power of the MDE on the species richness pattern. Simulated curves were based on empirical range sizes within a bounded domain, using the analytical stochastic models of Colwell and Hurtt (1994; Colwell & Lees, 2000). We conducted 100,000 Monte Carlo simulations of empirical range sizes sampled without replacement (i.e., the randomization procedure) to calculate the mean expected species richness and their 95% confidence intervals for each sampling site.

We applied two habitat indices, including vegetation type (VT) and community type (CT; Chen, 1958, 1963), and counted the total number of VT and CT to quantify habitat complexity for each sampling site. We in total categorized six VTs, including coniferous forest, broad‐leaved forest, bush wood, shrub grass, grass, and meadow, and four CTs, including aquatic community, stone community, soil community, and woody community. We recorded the VT for each sampling site and the CT occupied by each moss specimen.

We obtained climate data from the WorldClim v2 database (Fick & Hijmans, 2017) in 30‐arc‐second (c. 1 km^2^) digital maps, including solar radiation (SR), annual precipitation (AP), annual AT, and wind speed (WS). The data extraction was implemented in ArcGIS 10.2 (ESRI). Finally, the summary of environmental and richness data is shown in Table S2.

### 2.4 Data analyses

**FIGURE 1:**
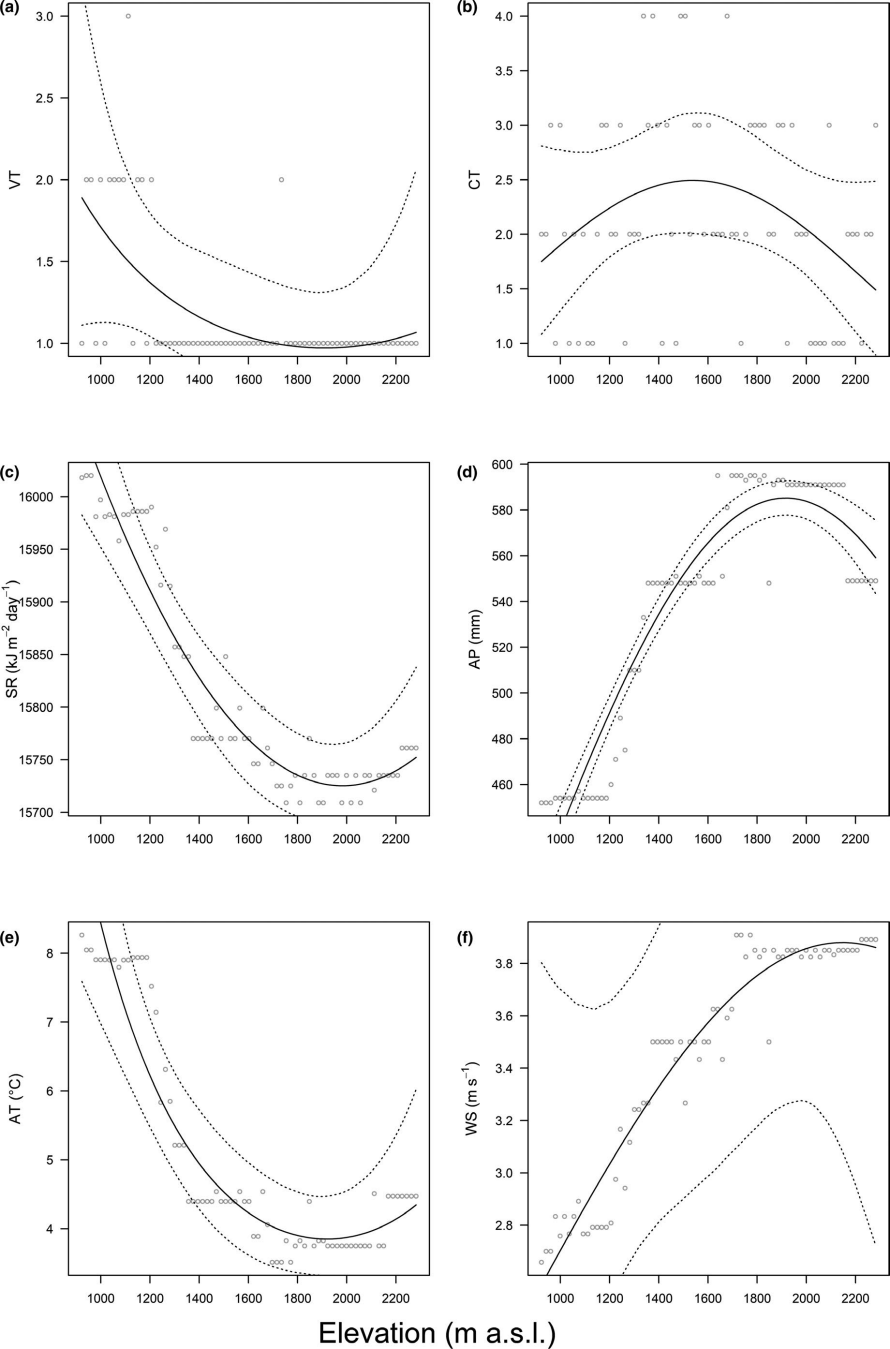
Elevational pattern of Mt Tuofeng for (a) vegetation type (VT), (b) community type (CT), (c) solar radiation (SR), (d) annual precipitation (AP), (e) average temperature (AT), and (f) wind speed (WS) with regression line (black line) and 95% credible interval (dotted lines). Hollow circles represent observed values

We applied polynomial regressions (PRs) to estimate the relationship between species richness and elevation, guided by the corrected Akaike information criterion (AICc) value. We used generalized linear models (GLMs) to evaluate the elevational pattern for each ecological predictor, to assess the relationship between moss species richness and habitat diversity, and to predict the occurrence probability of each moss family along the elevational gradient by using presence/absence as the dependent variable. Acknowledged that these relationships may not be linear, especially that the elevational gradient of species richness can take many shapes but most often takes a hump‐shaped pattern (Rahbek, Borregaard, Colwell, et al., 2019), we included polynomials of elevation up to the second degree for each GLM. We collected random samples from posterior distribution to estimate the 95% credible intervals for model parameters for the above models through the ARM package (Gelman & Su, 2016). The elevational trends of the predictors are shown in Figure 1. We fit multinomial models in a Bayesian framework using Markov chain Monte Carlo simulations in OpenBUGS through the package R2OpenBUGS (Sturtz et al., 2005), using elevation as the predictor and CT the outcome variable for the six most common moss families, respectively. Two Markov chains were simulated, each of length 10,000. The burn‐in was set to 1,000, and the chain was thinned by two to save work space and reduce autocorrelation. Convergence was assessed graphically and by the R‐hat value (Brooks & Gelman, 1998).

We used an information‐theoretic approach (Anderson et al., 2000; Chen et al., 2020; Stephens et al., 2005) to examine the relative roles of the MDE, the habitat complexity hypothesis, the energy hypothesis, and the environment hypothesis to moss species richness along the elevational gradient. Prior to analyses, all continuous variables (MDE, VT, CT, SR, AP, AT, and WS) were log‐transformed to achieve normality and homoscedasticity. Then, we used GLM to build possible candidate models based on a priori hypotheses. Owing to the complication of habitat complexity hypothesis and environment hypothesis, we establish the models that included all the possible combinations of two habitat‐related factors (VT and CT) for habitat complexity theory and three environment‐related factors (AP, AT, and WS) for environment theory. A null model (richness ~ 1) was added for comparison. We calculated the variance inflation factor (VIF) of each variable in each model to assess collinearity. To reduce multicollinearity, only the models with VIFs < 10 were considered (Chen et al., 2020; Dormann et al., 2013). We performed model averaging to evaluate the relative importance of each variable in shaping the elevational richness pattern (Galipaud et al., 2017). Last, we selected the best model through a forward stepwise selection algorithm. In the initial stage, we selected the model using a single variable with the minimum AICc. In the second stage, we then examined all two‐variable models that included the variable chosen in the first step and chose the model with the minimum AICc. We then repeated the procedure for all three‐variable models that included the two already selected, and so on, until AICc could not be further reduced. And this procedure was completed in the MuMIn package (Bartoń, 2015).

Thereafter, we used additive diversity partitioning to quantify the heterogeneity in biodiversity by comparing the diversity within (*α*) and among (*β*) sampling sites and by comparing the contributions made by elevation (*β*_elevation_) and other factors (*β*_replace_). In the additive approach, diversity can be explored across spatial scales (Gering & Crist, 2002), and γ‐diversity (regional scale) is partitioned into the sum of the average diversity of sampling sites (*α*) and the heterogeneity among sampling sites (*β*). When a species is missing from a sampling site, one reason might be that the sampling site is bearing more geometric constraints of montane topography according to the MDE. So, we used additive diversity partitioning combined with species richness pattern predicted by the MDE and quadratic polynomial regression, respectively, to partition *β* into *β*_elevation_, which represents the average difference between *α* and the maximum diversity predicted by the MDE or quadratic polynomial regression (*S*_max_) and *β*_replace_, the average number of missing species that are not explained by elevation. Because *α*, *β*, *β*_elevation_, *β*_replace_, and γ‐diversity are measured using the same units, their relative importance can be quantified (Crist & Veech, 2006). We performed all analyses using R 3.5.2 (R Development Core Team, 2018).

## 3 RESULTS

### 3.1 Species richness pattern

**FIGURE 2:**
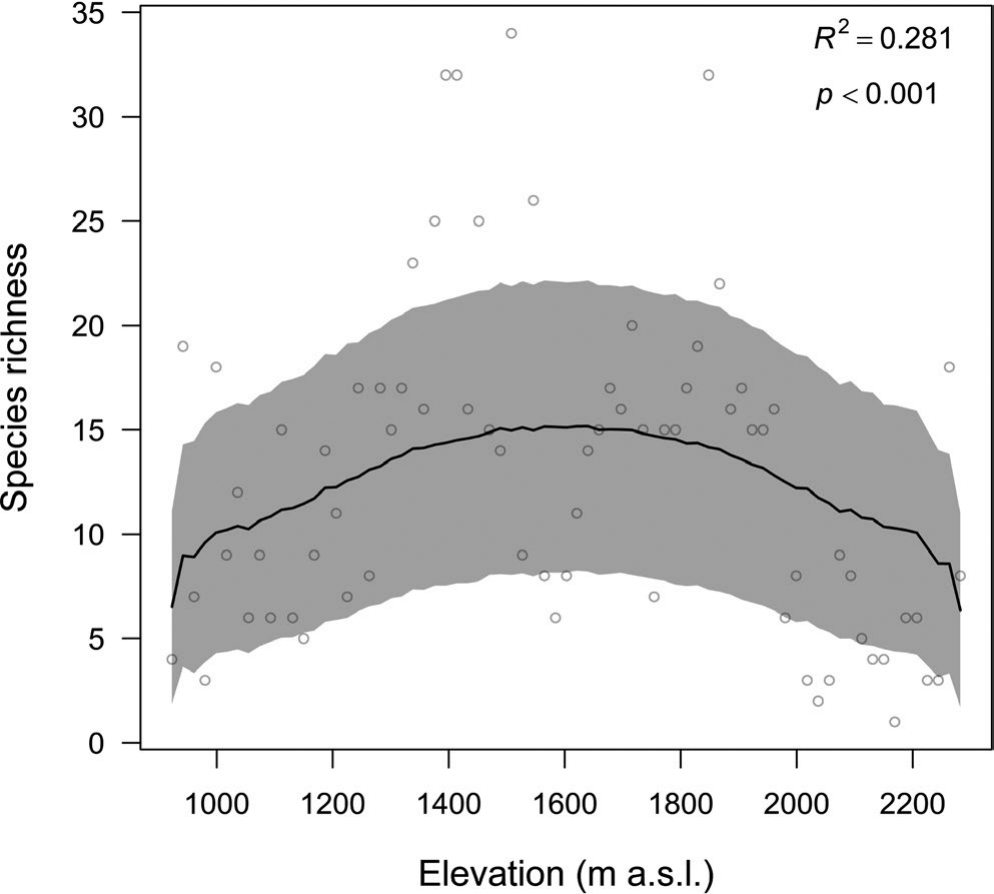
Relationship between the MDE and species richness. Hollow circles represent the species richness, and the black line is the predicted mean richness derived from RangeModel. Shaded areas show the 95% confidence interval of the prediction. The *R* ^2^ and *p*‐values were obtained by doing a linear regression of the observed richness on the predicted values to estimate the impact of the null model

**FIGURE 3:**
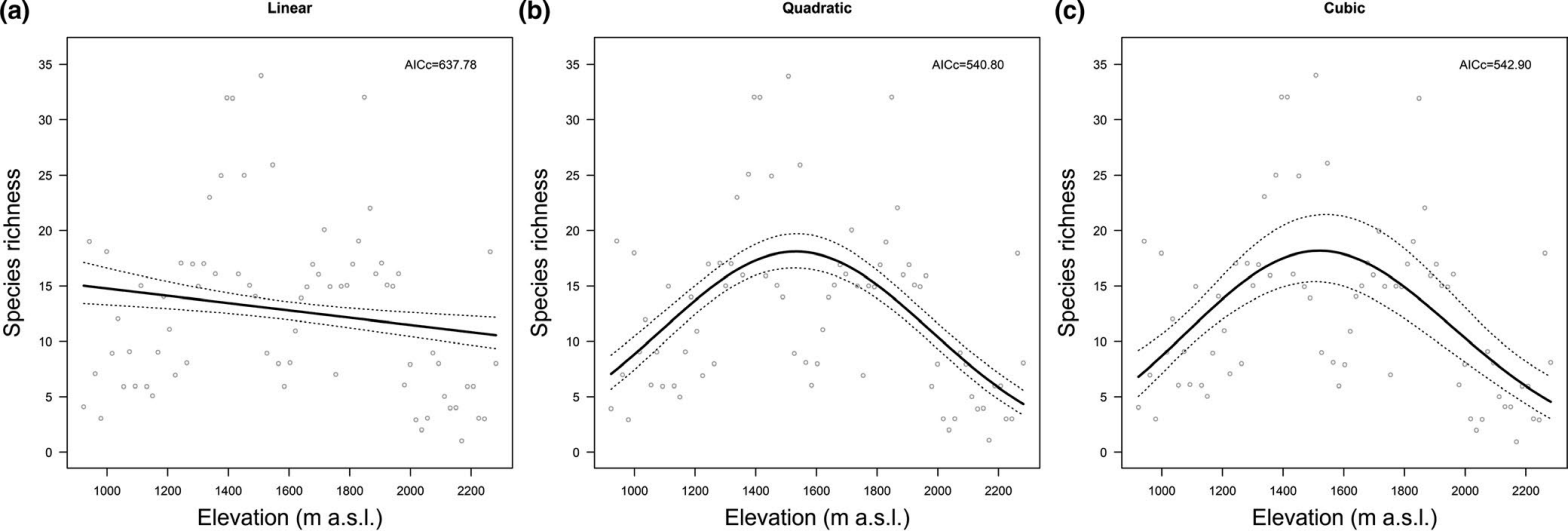
Polynomial regressions of moss species richness along elevational gradient with regression line (black line) and 95% credible interval (dotted lines). Hollow circles represent observed values

A total of 191 moss species, belonging to 73 genera under 26 families, were identified in 1,301 specimens at 73 sampling sites along the elevational gradient, in which four species (*Drummondia sinensis*, *Sanionia uncinate*, *Tayloria indica*, and *Funaria hygromexrica*) are endemic. Pottiaceae (41 species), Brachytheciaceae (35 species), Bryaceae (20 species), Entodontaceae (12 species), Hypnaceae (10 species), and Grimmiaceae (nine species) are the most common moss families taking up to 66% of the species composition (Table S3). Moss species in the Mt Tuofeng showed a hump‐shaped richness pattern along the elevational gradient, with a distinct peak at 1,500 and 1,600 m a.s.l. (Figure 2). This result was confirmed by the polynomial regressions, where the quadratic polynomial regression (AICc = 540.80) with a hump‐shaped pattern performed best (Figure 3). Among the six VTs and four CTs, broad‐leaved forest and stone community are the most species‐rich VT and CT, harboring 155 and 121 species, respectively (Table 1). All four communities were recorded in five sampling sites with an elevation range between 1,338 and 1,678 m a.s.l. (Table S2), corresponding to the species richness peak.

**TABLE 1:** Taxonomic distribution within each vegetation and community type

Taxonomic rank	Vegetation type						Community type			
	CF	BF	BW	SG	G	M	A	ST	SO	W
Family	20	22	22	16	10	13	18	21	18	23
Genus	42	64	60	34	22	22	49	58	47	53
Species	65	155	109	56	40	43	100	121	100	98

Abbreviations: A, aquatic community; BF, broad‐leaved forest; BW, bush wood; CF, coniferous forest; G, grass; M, meadow; SG, shrub grass; SO, soil community; ST, stone community; W, woody community.

### 3.2 Relationship between species richness and explanatory factors

The information‐theoretic statistics for the nine candidate models showed that MDE was suggested as the best model, which had an Akaike weight (*W*_i_) of 0.98 and explained a largest proportion of variation for moss species richness pattern (*R* ^2^ = 0.245, *p* < 0.001; Table 2). Two alternative habitat‐related models also provided a significant proportion of variation, one including only CT (*R* ^2^ = 0.146, *p* < 0.001) and the other including CT and VT (*R* ^2^ = 0.135, *p* < 0.01), but their ΔAICc exceeds two. The null model (richness ~ 1) had little support to the species richness pattern (ΔAICc = 19.33, *W*_i_ = 0.00). The best model for our dataset was the combination of MDE and CT, which reduced the AICc from 490.16 for MDE alone to 484.32 for MDE and CT, suggesting the MDE and the habitat complexity hypothesis were supported (Table 3).

**TABLE 2:** Results of candidate models explaining variation for moss species richness pattern in the Mt Tuofeng

Hypothesis	Model	*K* ^a^	Adjust *R* ^2^	AICc	ΔAICc	AICc *W*_i_
Null model	Richness ~ 1	2	n.a.	509.49	19.33	0.00
Mid‐domain effect	MDE	3	0.245***	490.16	0.00	0.98
Habitat complexity	VT	3	−0.006	511.05	20.89	0.00
	CT	3	0.146***	499.15	8.99	0.01
	CT + VT	4	0.135***	501.28	11.12	0.00
Energy	SR	3	−0.004	510.92	20.76	0.00
Environment	AP	3	0.005	510.25	20.09	0.00
	AT	3	0.017	509.39	19.23	0.00
	WS	3	−0.013	511.57	21.41	0.00

Abbreviations: AP, annual precipitation; AT, annual average temperature; CT, community type; MDE, the mid‐domain effect; SR, solar radiation; VT, vegetation type; WS, wind speed.

^a^ Number of estimable parameters.

** *p* < 0.01.

*** *p* < 0.001.

**TABLE 3:** Final model selection for candidate explanatory variables for moss species richness pattern in the Mt Tuofeng

Hypothesis	Model	Slope	Adjust *R* ^2^	AICc
Mid‐domain effect	1. MDE	5.35	0.245	490.16
Habitat complexity	2. CT	9.55	0.069	484.32
	Final model		0.314	484.32

Note

Variables that entered each model are numbered in order of entry. Slope is the standardized partial regression slope of the variable in the final model. For the numbered variables, adjust *R* ^2^ is the increase in total variance explained as each variable entered the model. For the final model, adjust *R* ^2^ is the total explained variance for the final model. AICc is the corrected Akaike information criterion for each model.

Abbreviations: CT, community type; MDE, the mid‐domain effect.

### 3.3 Additive partitioning of diversity

**FIGURE 4:**
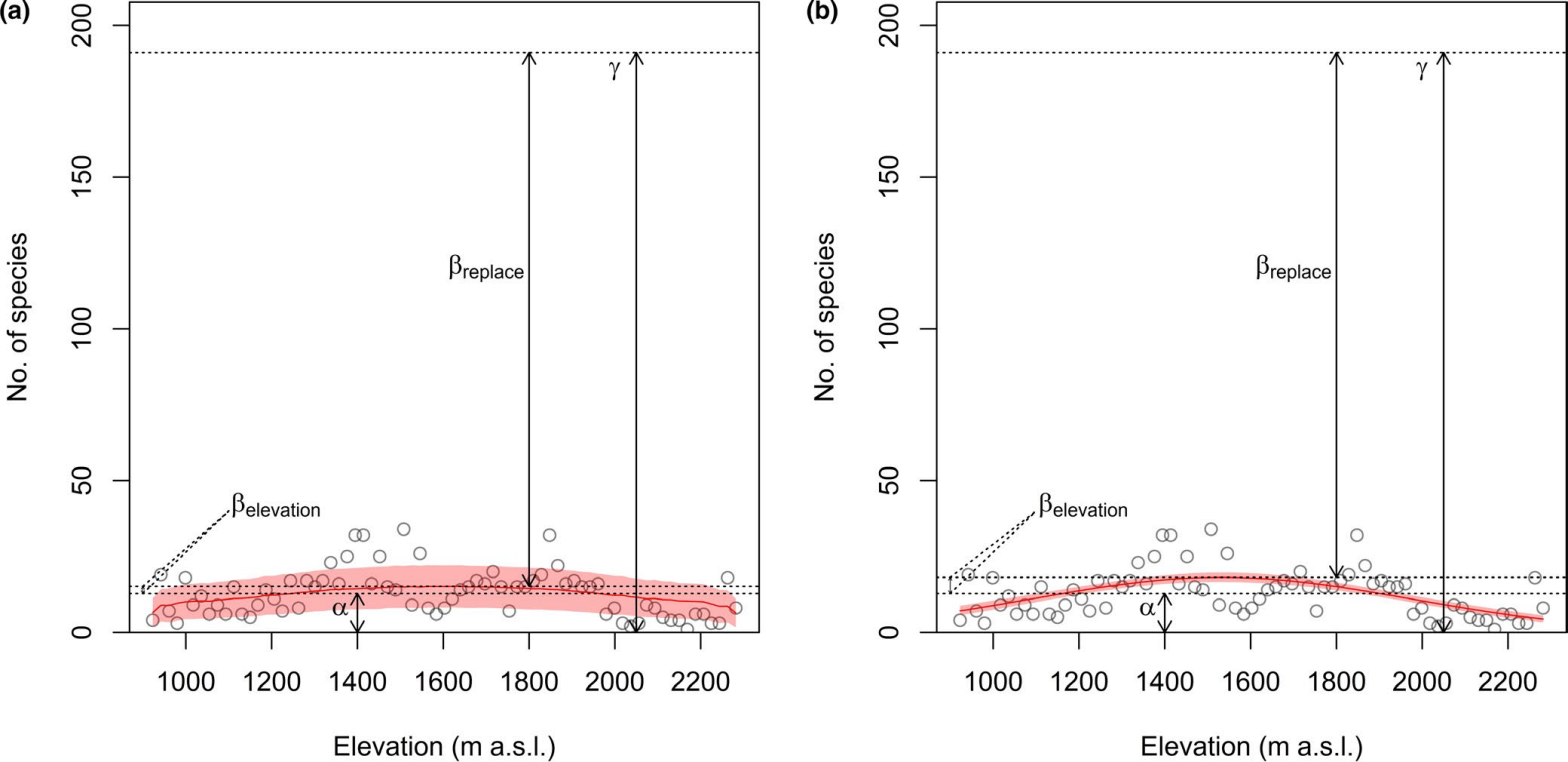
Combination of additive diversity partitioning and the relationship between species richness and elevation predicted by (a) the mid‐domain effect and (b) quadratic polynomial regression, showing α‐, β‐, and γ‐diversity. *β* is partitioned into *β*_elevation_ (contributions made by elevation) and *β*_replace_ (contributions made by other factors). The solid red line represents the MDE null predicted line in (a) or quadratic polynomial regression line in (b). Shaded areas show the 95% confidence interval of the prediction in (a) or 95% model credible interval in (b)

According to the additive diversity partitioning, *α* (12.79) explained only 6.7% of the variation in species richness, whereas *β* (178.21) explained about 93.3% of the variation in species richness (Figure 4).

We calculated the contribution of elevation toward the variation in species richness by using MDE prediction and quadratic polynomial regression, respectively. The contribution of elevation ranged from 44.6% in the MDE prediction (Figure 4a) to 53.3% in the quadratic polynomial regression (Figure 4b), with an average of 49.0% toward the variation in species richness.

We also compared *β*_elevation_ and *β*_replace_ through MDE prediction and quadratic polynomial regression, respectively. The proportion of *β*_elevation_ to the total *β* ranged from 1.3% in the MDE prediction (Figure 4a) to 3.0% in the quadratic polynomial regression (Figure 4b), with an average of 2.2%. The proportion of *β*_replace_ to the total *β* was 97.8% on average, about 44 times the proportion of *β*_elevation_.

### 3.4 Occurrence probability and proportional use of community type along the elevational gradient

Occurrence probability along the elevational gradient varied among the 26 moss families (Figure S1; Figure 6). Bartramiaceae, Bryaceae, and Ditrichaceae decreased first and then increased with elevation; Ptychomitriaceae increased with elevation; and as for the other 22 moss families, occurrence probability increased first and then decreased with elevation, displaying a hump‐shaped pattern, among which, occurrence probability peaked below the mid‐elevation for Amblystegiaceae, Anomodontaceae, Drummondiaceae, and Orthotrichaceae, above the mid‐elevation for Fabroniaceae, Funariaceae, Scorpidiaceae, and Splachnaceae, and around the mid‐elevation for Brachytheciaceae, Encalyptaceae, Entodontaceae, Fissidentaceae, Grimmiaceae, Hypnaceae, Leskeaceae, Leucodontaceae, Mniaceae, Plagiotheciaceae, Pottiaceae, Pseudoleskeellaceae, Pylaisiaceae, and Thuidiaceae (Figure S1).

Proportional use of CT along the elevational gradient varied among the six most common moss families (Figure 7). As the increase of elevation, Brachytheciaceae, Bryaceae, and Pottiaceae increased the proportional use of woody community, whereas Entodontaceae, Grimmiaceae, and Hypnaceae basically maintained constant; Brachytheciaceae, Bryaceae, Entodontaceae, and Pottiaceae decreased the proportional use of soil community, whereas Grimmiaceae and Hypnaceae basically maintained constant; Grimmiaceae decreased, whereas the other families increased the proportional use of aquatic community; and as for stone community, Hypnaceae and Pottiaceae decreased, Entodontaceae and Grimmiaceae increased, whereas Brachytheciaceae and Bryaceae maintained constant (Figure 7).

## 4 DISCUSSION

### 4.1 Species richness pattern of mosses on the Mt Tuofeng

**FIGURE 5:**
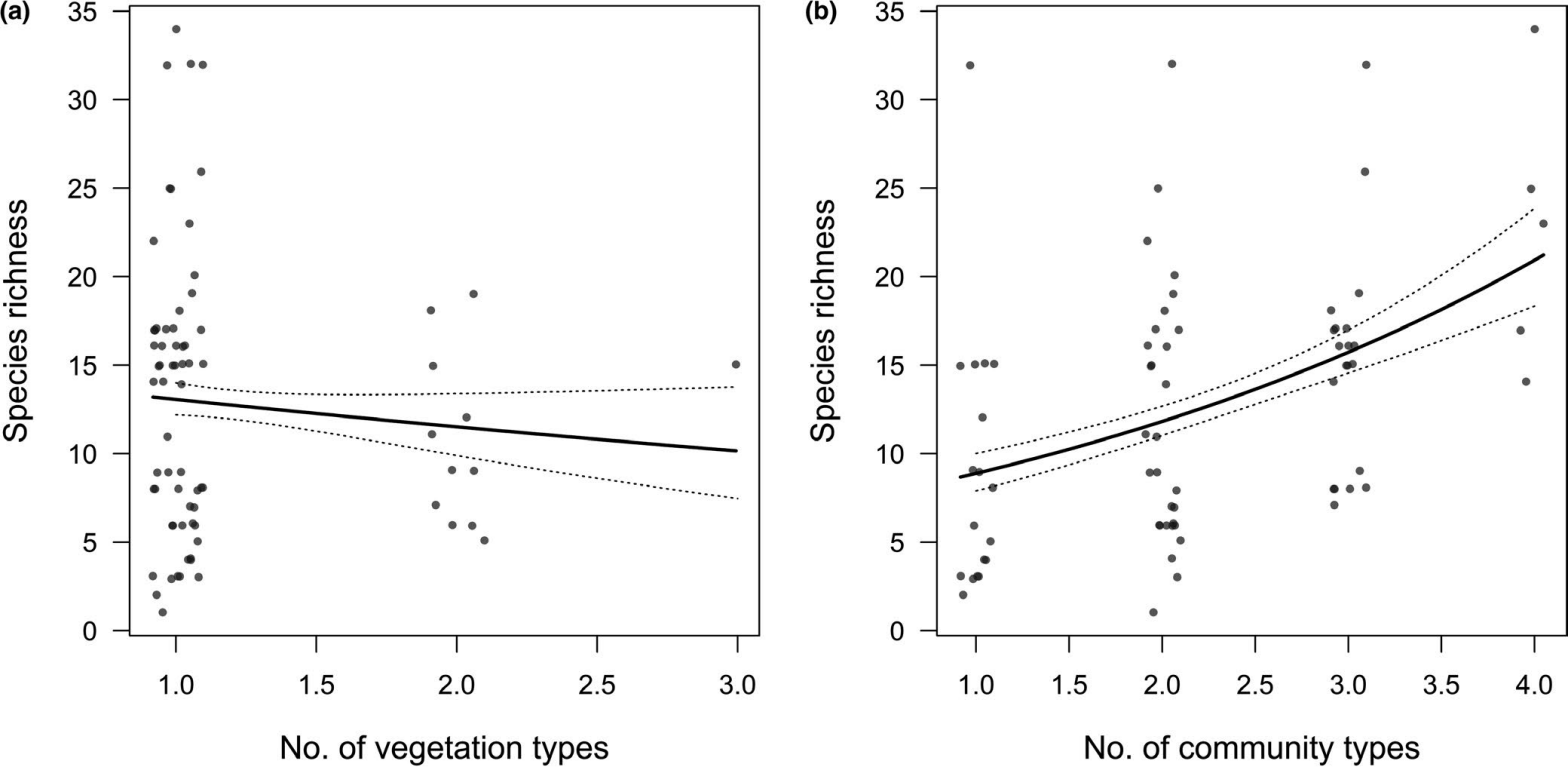
Moss species richness versus (a) the number of vegetation types and (b) the number of community types with regression line and 95% credible interval (dotted lines). Black dots represent observed value that jittered in the horizontal direction

The overall moss species richness pattern along the elevational gradient on the Mt Tuofeng is a hump‐shaped pattern, peaking at mid‐elevation between 1,500 and 1,600 m a.s.l., consistent with some studies in moss species (Grau et al., 2007; Wolf, 1993). However, in other studies, moss species richness was found to have either no statistically significant trend (Grytnes et al., 2006; Sun et al., 2013) or an increasing trend (Bruun et al., 2006) with altitude. The hump‐shaped richness pattern is popular in studies from around the world, but there is no consistent explanation for this pattern. Our analysis showed that the MDE and the habitat complexity hypothesis concur to the elevational moss species richness. Model selection among alternative models showed that MDE is the primary driver for richness patterns, whereas little support was found for the energy and the environment. It is not surprising that the energy hypothesis and the environment hypothesis are not supported in our data, as moss species are characterized by their poikilohydric condition and cold tolerance. The cuticle that seals the vascular plants body is often reduced or even lacking on the gametophyte of mosses, making mosses tolerant of desiccation and poikilohydric, which means that their water content is directly regulated by ambient humidity (Proctor et al., 2007). Moreover, a common feature among most mosses is their ability to grow at low temperature, and studies have showed that subglacial bryophytes following up to six centuries of ice entombment successfully regenerate (Cannone et al., 2017; La Farge et al., 2013; Roads et al., 2014). These ecophysiological features enable them to grow on rocks and tree trunks that are inhospitable for most vascular plants, thereby reducing the impact of energetic and environmental factors. The habitat complexity hypothesis was supported because habitat diversity plays an important role in maintaining biodiversity, and the removal of habitat types will obliterate species, especially habitat specialists (Sfenthourakis & Triantis, 2009). What's more, the CT pattern along the elevational gradient on the Mt Tuofeng is also hump‐shaped (Figure 1b), implying a positive correlation between CT and moss species richness, which is proved by the GLM analysis (Figure 5b). A possible reason for the good support of MDE in our study is the high dispersal capacity of moss species (Patiño & Vanderpoorten, 2018), which makes a wide distributional range for most moss species, resulting in a high degree of overlap in the central area.

However, the drivers of elevational moss species richness pattern in our study are inconsistent with the studies of bryophyte diversity conducted by Raabe et al. (2010) and Spitale (2016) in European mountains where climate factors, such as temperature and SR, were the most important predictors. There are two reasons for this discrepancy. First, mosses and liverworts were sampled and analyzed in their studies. However, liverworts are usually more sensitive to drought than mosses (Oliver et al., 2000), so the drivers of elevational species richness pattern may differ between the two groups, resulting in a disguised or biased pattern of mosses. Second, in their studies bryophytes were sampled from soil and wood, whereas we sampled mosses not only associated with soil and wood but also from dry rocks and stream water. Because humidity is higher on the forest floor than on tree trunks (Proctor & Tuba, 2002), bryophyte assemblages inhabiting deadwood and tree trunks are mostly subject to climatic variability (Spitale, 2016). Therefore, the sampling choice in our study could buffer the impact of climate.

### 4.2 Three pieces of evidence support the MDE

In the tangled complexity of environmental and nonenvironmental factors affecting diversity gradients, new null models of the MDE helped to pare down the complexity, which is predicted where landmass boundaries such as oceans and mountaintops limit species ranges and the simple overlap of many, variously sized ranges create a peak in species richness at mid‐elevation (Colwell & Hurtt, 1994; Colwell & Lees, 2000). In our dataset, we found three evidence conforming to the MDE. First, moss species richness peaked at mid‐elevation (Figure 2). Second, within the 26 moss families, taxonomic groups that have a wider distribution usually peaked at mid‐elevation, whereas those have a narrower distribution usually peaked somewhere away from the middle according to their occurrence probability along the elevational gradient (Figure S1). Third, based on the edge effect or community overlap hypotheses, the greatest species richness exists in the ecotone areas of overlap between two distinct biological communities (Lomolino, 2001). However, in our case, we found moss species richness had a negative relationship with the number of occupied VT (Figure 5a), implying the interior portion of the floral unit harbors the highest species richness, given the fact that the higher WS and lower humidity at ecotone areas are adverse to moss species survival (Liu et al., 2007). Therefore, our result, on the contrary, supports the MDE.

### 4.3 How could elevation contribute to the heterogeneity

**FIGURE 6:**
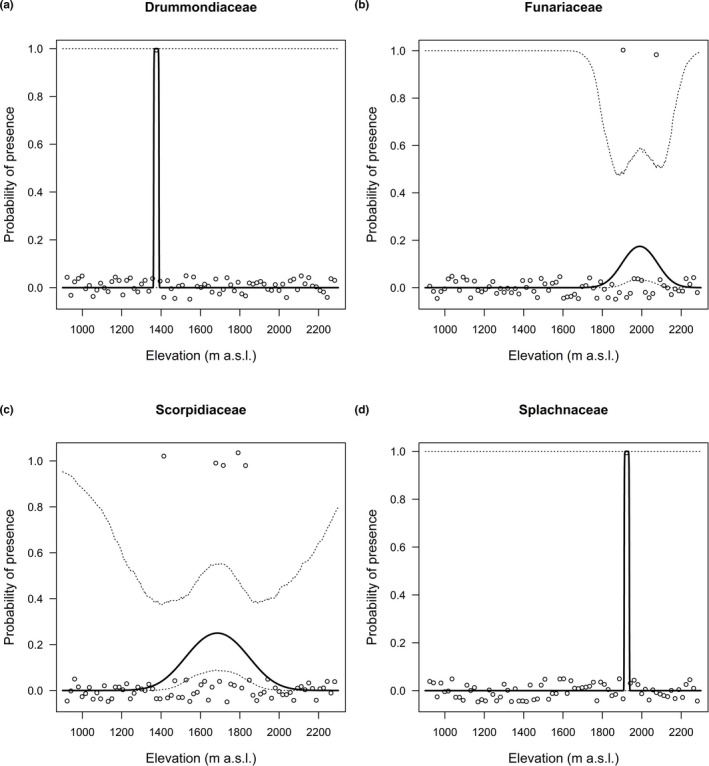
Moss presence data versus elevation with regression line and 95% credible interval (dotted lines) for four families that formed small‐ranged endemism. Open circles represent observed presence (1) or absence (0) that jittered in the vertical direction

**FIGURE 7:**
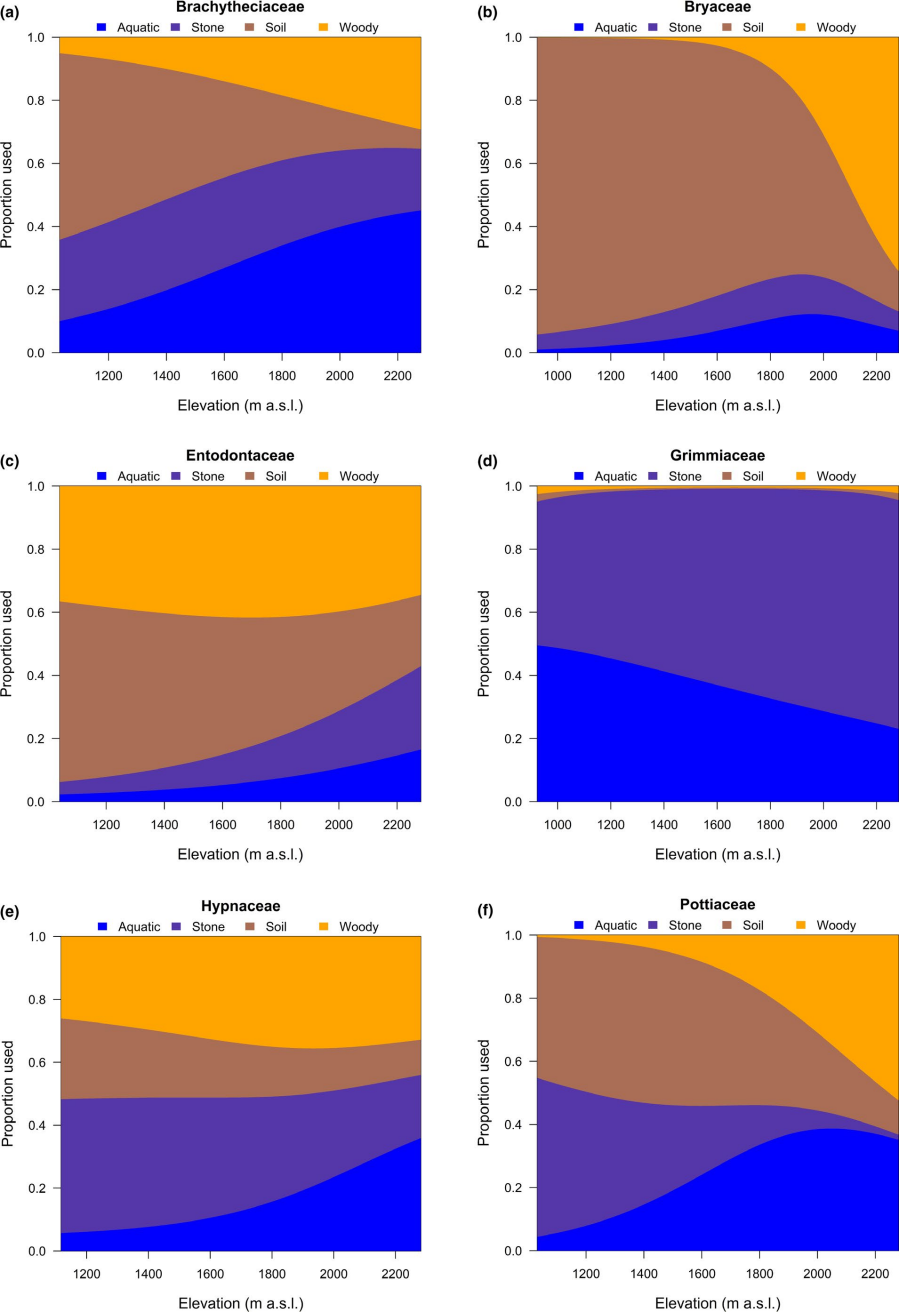
Proportional use of four community types in relation to elevation by (a) Brachytheciaceae, (b) Bryaceae, (c) Entodontaceae, (d) Grimmiaceae, (e) Hypnaceae, and (f) Pottiaceae

Elevation contributed toward explaining the heterogeneity, likely because of two facts. On one hand, the elevational gradient could provide heterogeneous environments (Hoorn et al., 2018; Rahbek, 1995), and different taxonomic groups survive at different elevation by selecting different physical conditions (Letten et al., 2013; Figure 6; Figure S1). On the other hand, the elevational gradient may enable habitat segregation among moss species. Indeed, sympatric taxonomic species sharing similar resources should demonstrate some degree of niche overlap, leading to interspecific competition (Chesson, 2000; Dufour et al., 2015). In turn, to buffer competition and allow for coexistence, sympatric species may avoid each other in space and/or time and can generate differences in habitat selection (Holt, 1987; Milleret et al., 2018). Proportional use of CT along the elevational gradient varied among moss families (Figure 7), reflecting a certain degree of habitat segregation. This strategy of habitat use may reduce the effect of competition among different taxonomic groups. Although this phenomenon was found at the family level, it gives insights into the behavior of the 191 species in the system. And we speculate that habitat segregation among species may be more significant than among families. Both the two facts associated with elevation help to explain why species assembly varies among sampling sites, contributing the heterogeneity explained by elevation.

### 4.4 Elevation had a very limited contribution toward explaining heterogeneity

As many as 34 species have been identified on a single sampling site, however, α‐diversity explained only 6.7% of γ‐diversity. The small *α* (12.79) indicates low species evenness, for the degree of species overlap varied across the elevational gradient. Elevation explained 49.0%, a medium predictive power, for the variation of species richness. Because the unique ecophysiological features of mosses make them likely independent of factors that associated with elevation (Figure 1), such as temperature, rainfall, and humidity which usually well govern the species richness of other taxa (Heaney, 2001; McCain, 2007; Sánchez‐Cordero, 2001), thereby lowering the *S*_max_ predicted either by the MDE or quadratic polynomial regression (Figure 4). Moreover, elevation explained 2.2%, a weak predictive power, for the heterogeneity, and this may be due to four facts. First, according to the calculation formula (*β*_elevation_ = *S*_max_ − *α*), a lower predicted *S*_max_ will further reduce the *β*_elevation_. Second, elevation or factors highly associated with elevation cannot adequately capture the high spatial heterogeneity of ecological and environmental variable characteristic of mountains. Third, mountain regions are home to aggregations of small‐ranged species which could form centers of endemism. Fourth, moss species are supposed to have a high dispersal capacity; however, the large *β*_replace_ in our study implies a high extinction rate when they colonize a new locality, as can be seen from Table S1 that many moss species appear discretely from sampling sites despite their wide distribution.

To conclude, current species richness distribution pattern may bear the signatures of ecological and evolutionary effects, whereas evolutionary factors predominately shape the large heterogeneity through dispersal, extinction, and speciation processes. To further explore the extent to which each factor shapes the current pattern, we agree with Rahbek, Borregaard, Antonelli, et al. (2019) that geological and evolutionary approaches should be combined for an accurate reconstruction of geological dynamics and reliable inference of the timing and location of changes in effective population sizes and genetic bottlenecks.

### 4.5 Applicability to biodiversity conservation

The practical importance of these results for conservation is threefold. First, the positive relationship between species richness and CTs suggests habitat diversity is essential for sustaining species diversity, so conservation of habitat diversity is the key to maintain moss species diversity in the mountain. Second, the unimodal richness pattern we detected suggests that the highest moss species richness appears at mid‐elevation; however, due to the large β‐diversity and very small *β*_elevation_, conservation efforts should be paid to the whole elevational range rather than the mid‐elevation only. Last, species of the moss families that have a very narrow distribution along the elevational gradient such as Drummondiaceae, Funariaceae, Splachnaceae, and Scorpidiaceae are likely to form small‐ranged endemism (Figure 6). And special attention should be paid to preserve these irreplaceable species according to their distribution.

## CONFLICT OF INTEREST

The authors declare there are no competing interests.

## AUTHOR CONTRIBUTIONS

**De Gao:** Conceptualization (equal); Formal analysis (equal); Investigation (equal); Writing‐original draft (lead); Writing‐review & editing (equal). **Liqin Fu:** Data curation (equal). **Jiaxing Sun:** Data curation (equal); Investigation (equal); Writing‐review & editing (equal). **Yan Li:** Data curation (equal); Investigation (equal); Writing‐review & editing (equal). **Zhen Cao:** Conceptualization (equal); Formal analysis (equal); Writing‐review & editing (equal). **Yongying Liu:** Investigation (equal). **Peng Xu:** Data curation (equal). **Jiancheng Zhao:** Investigation (equal); Writing‐review & editing (equal).

## Supporting information

Figure S1

Table S1

Table S2

Table S3

Supplementary Material

## Data Availability

All datasets used in this study are sourced from the literature which can be found in Supporting Information.
