# Supplementary material for: The mid‐domain effect and habitat complexity applied to elevational gradients: Moss species richness in a temperate semihumid monsoon climate mountain of China
Source: Ecol Evol. 2021 May 4;11(12):7448–60. doi: 10.1002/ece3.7576 (PMC8216932; doi:10.1002/ece3.7576)
Supplement: Supplementary file 1 — Figure S1 [file ECE3-11-7448-s003.docx]

**Figure S1.** Moss presence/absence data versus elevation with regression line (black line) and 95% credible interval (dotted lines) for 26 families listed in alphabetical order. Open circles represent observed presence (1) or absence (0) that jittered in the vertical direction.

| 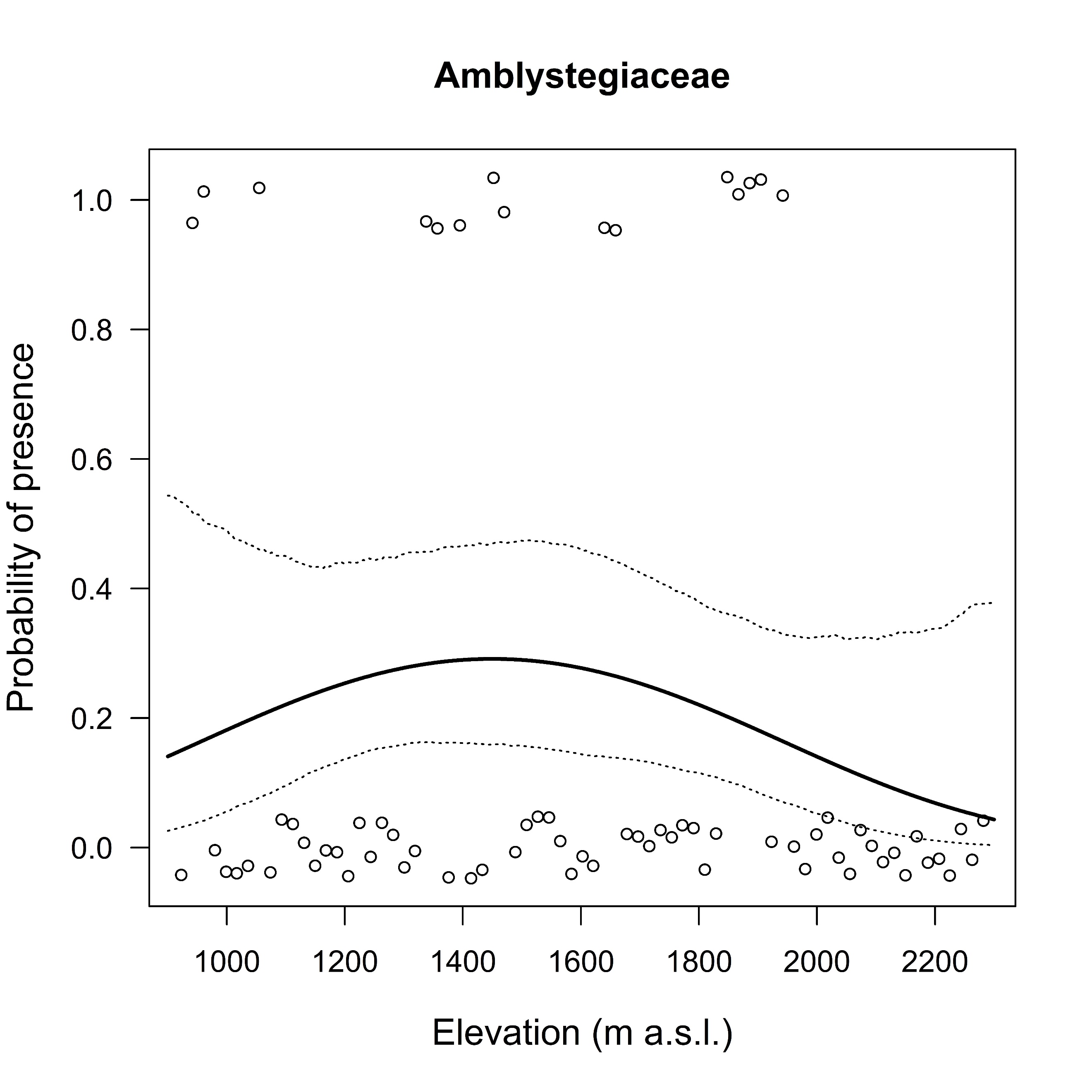 | 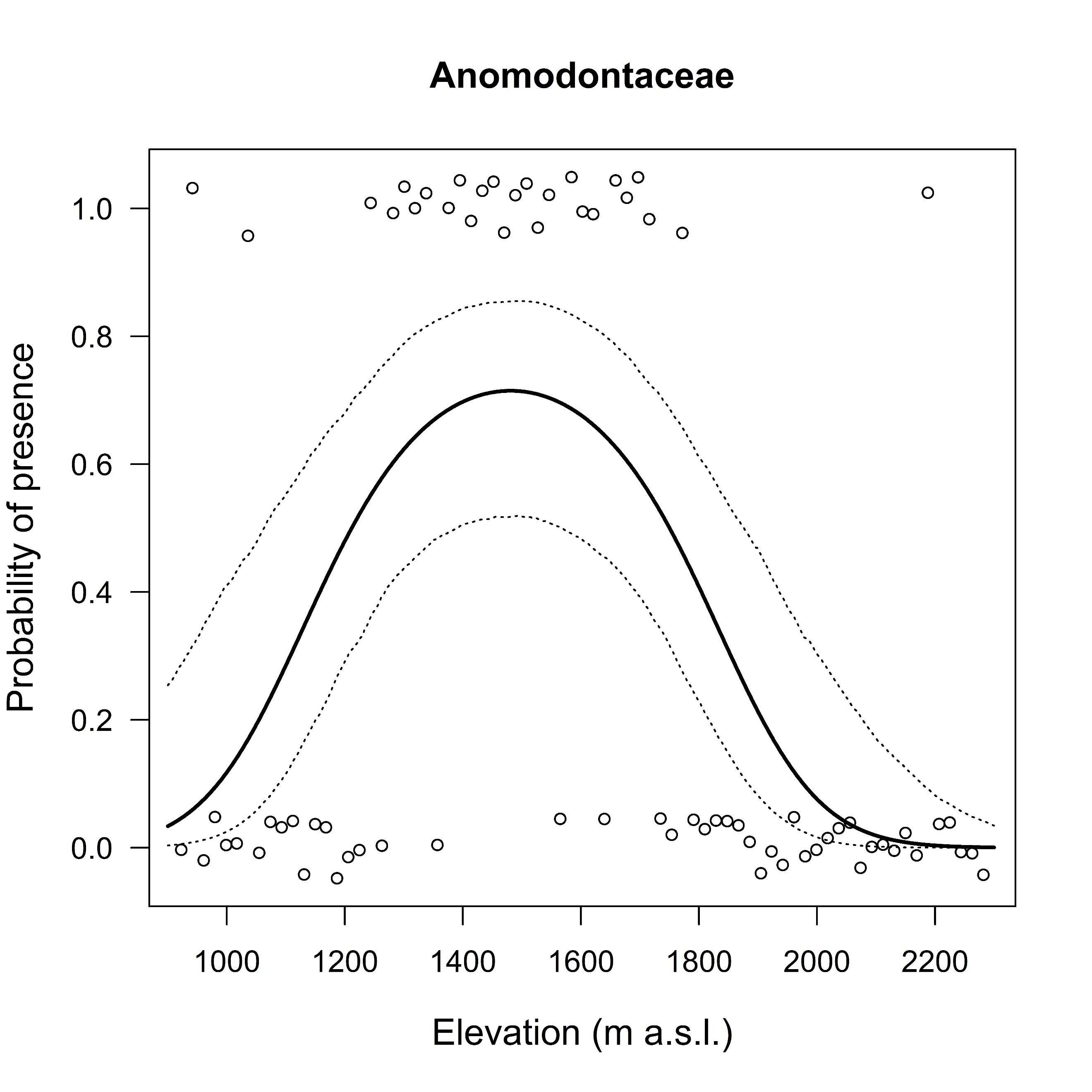 |
| --- | --- |
| 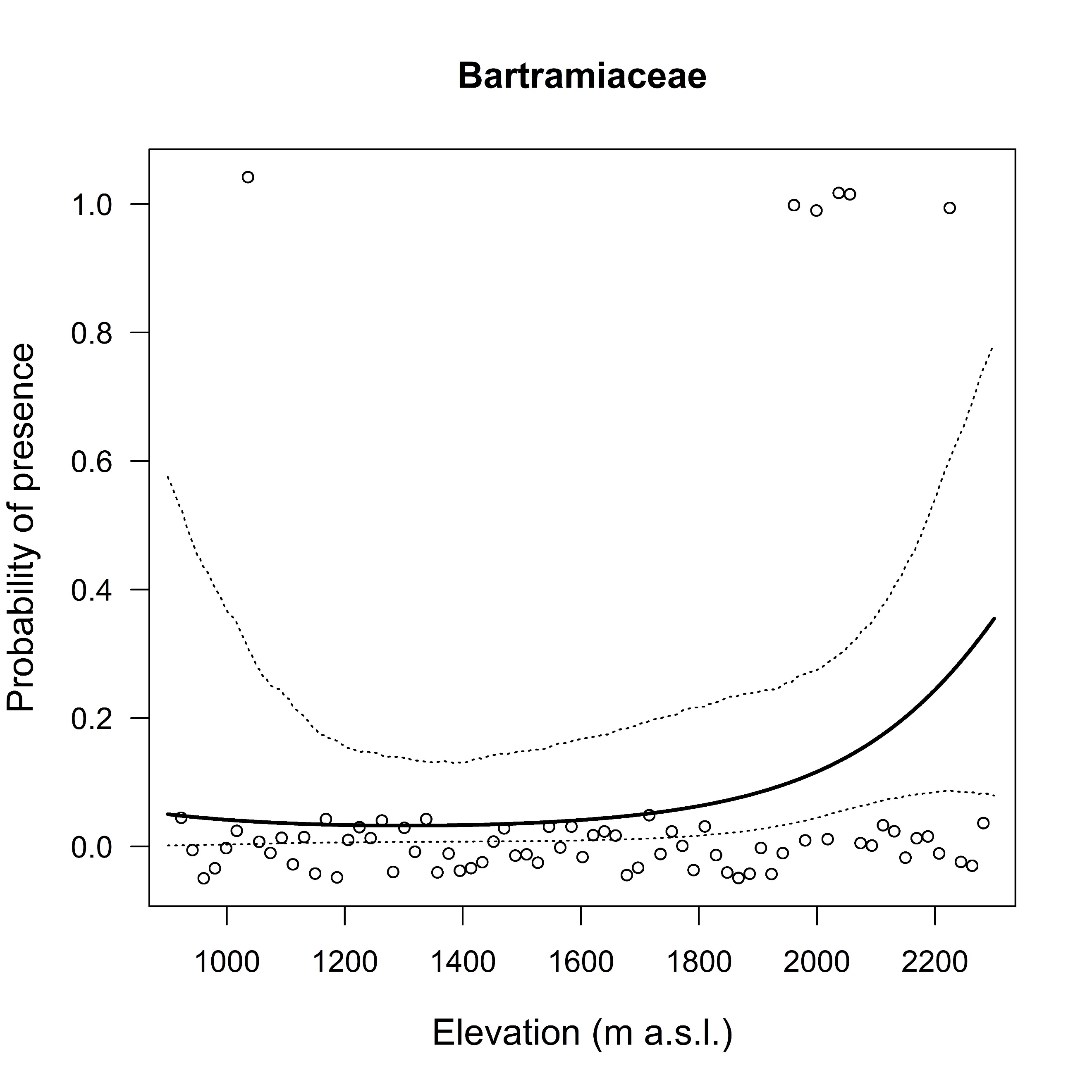 | 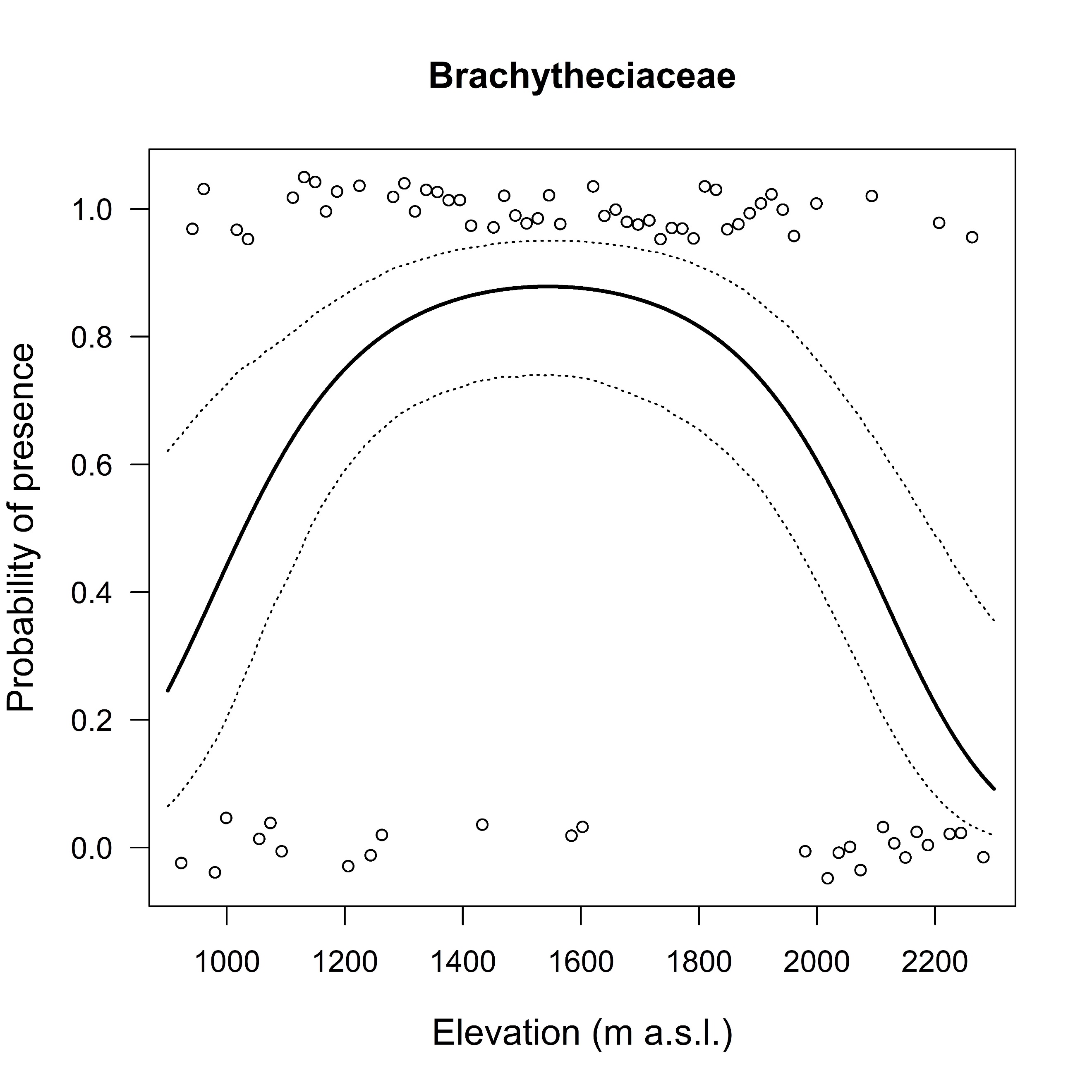 |
| 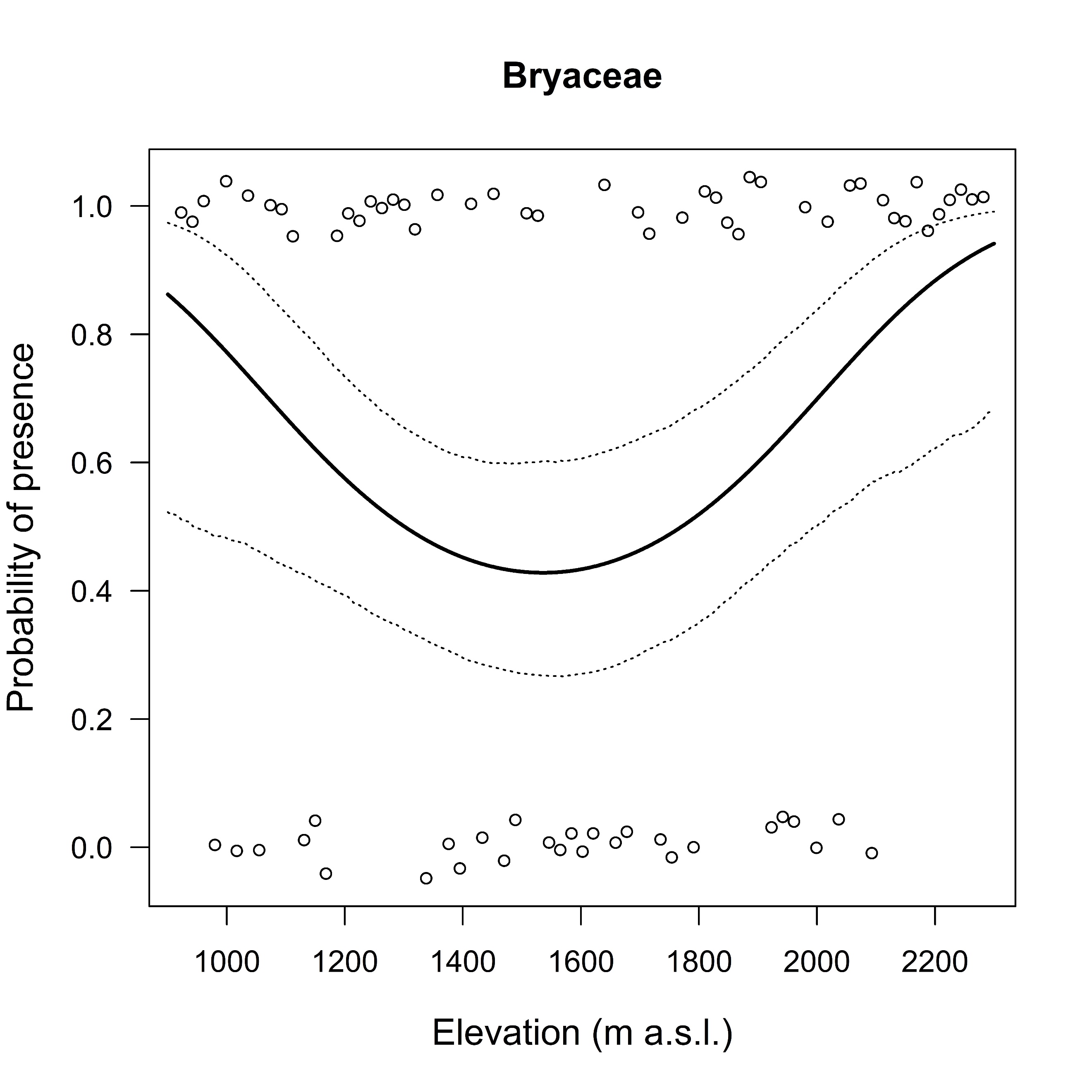 | 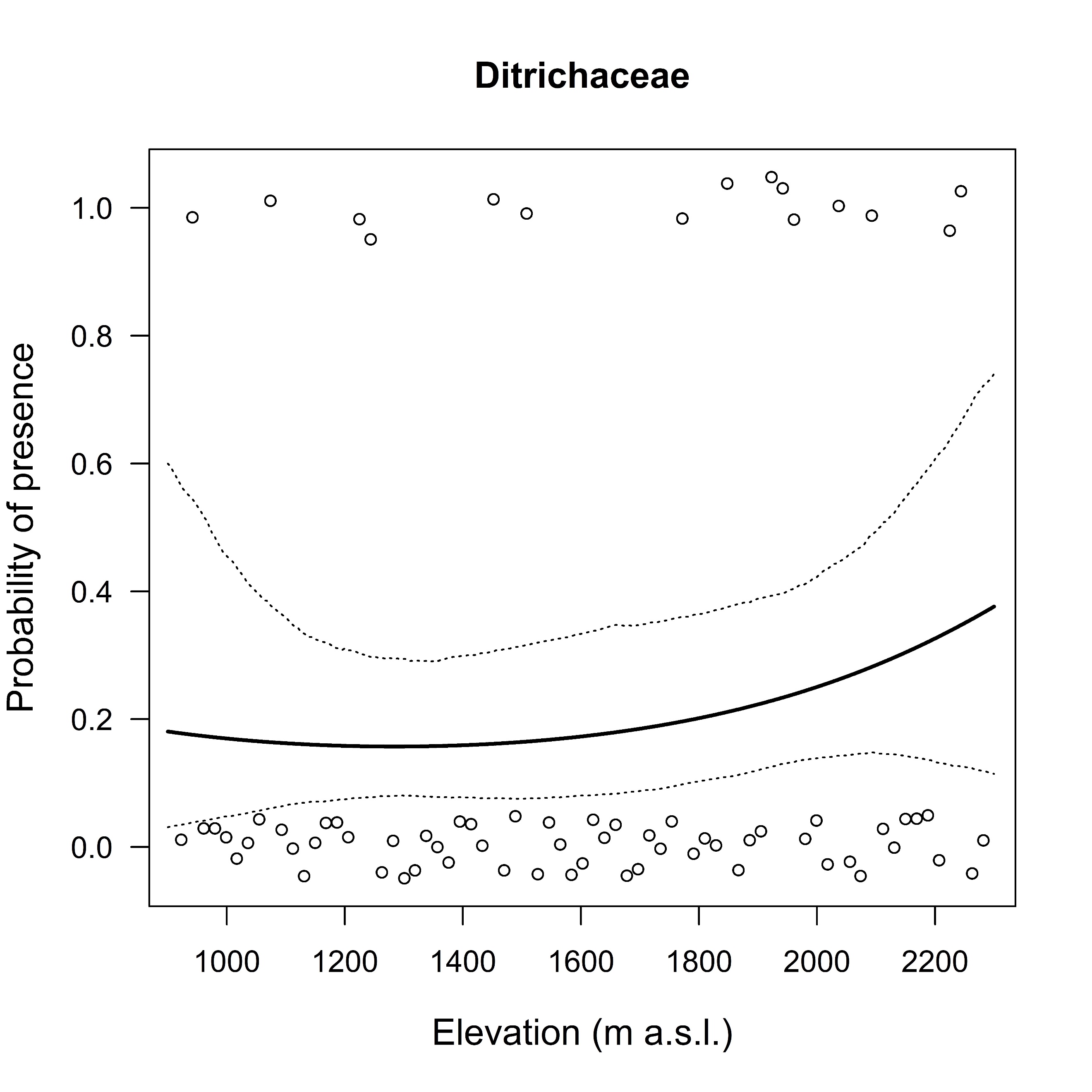 |
| 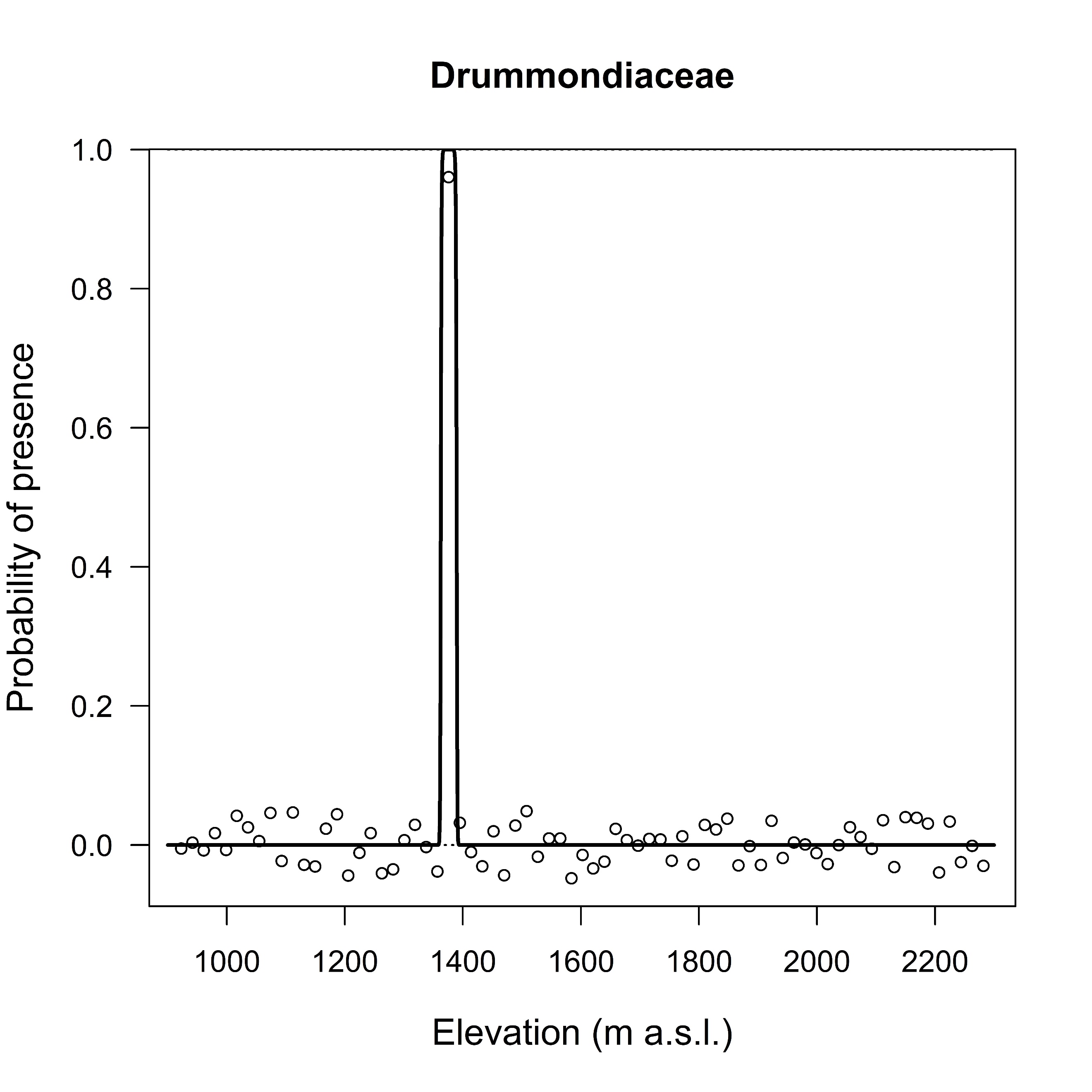 | 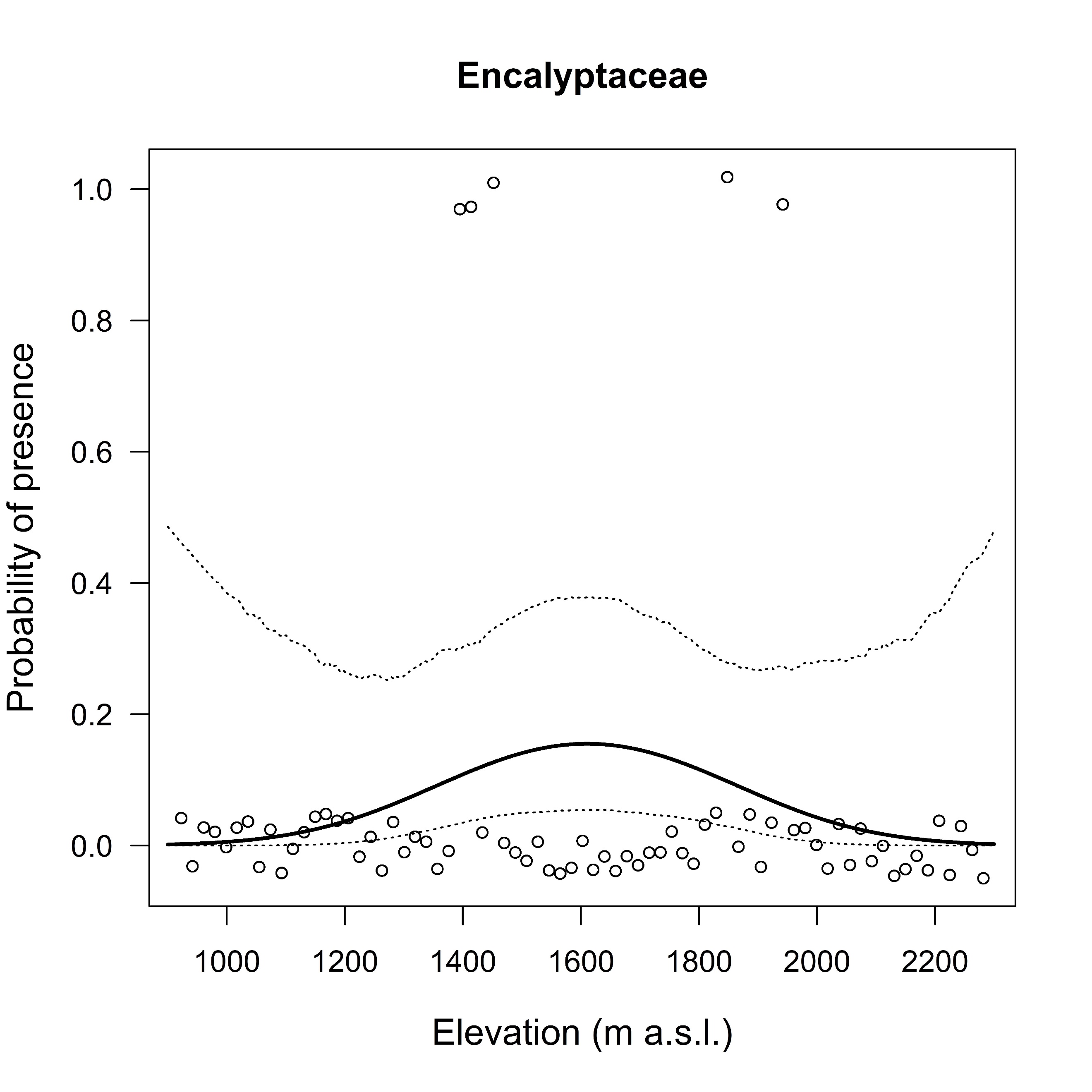 |
| 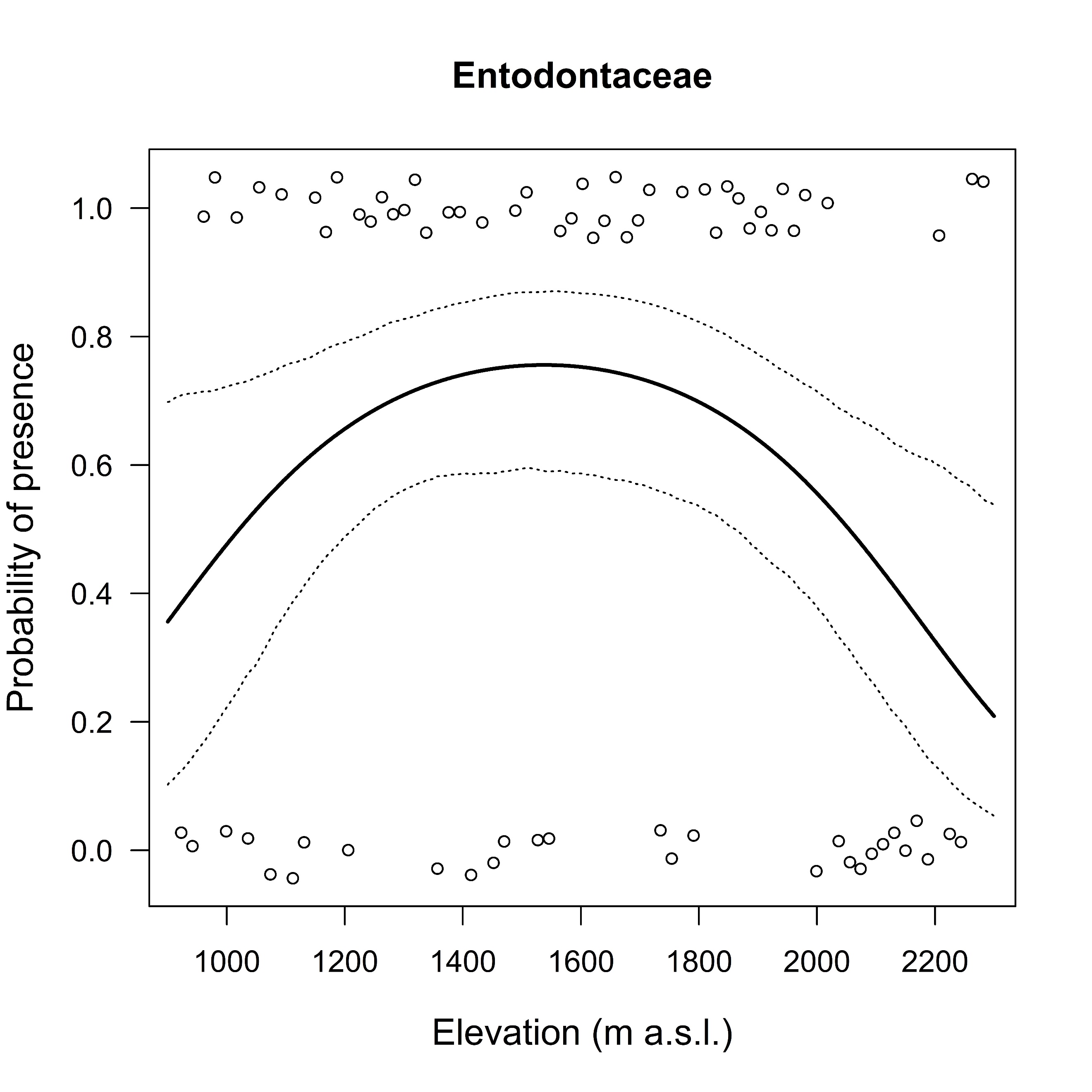 | 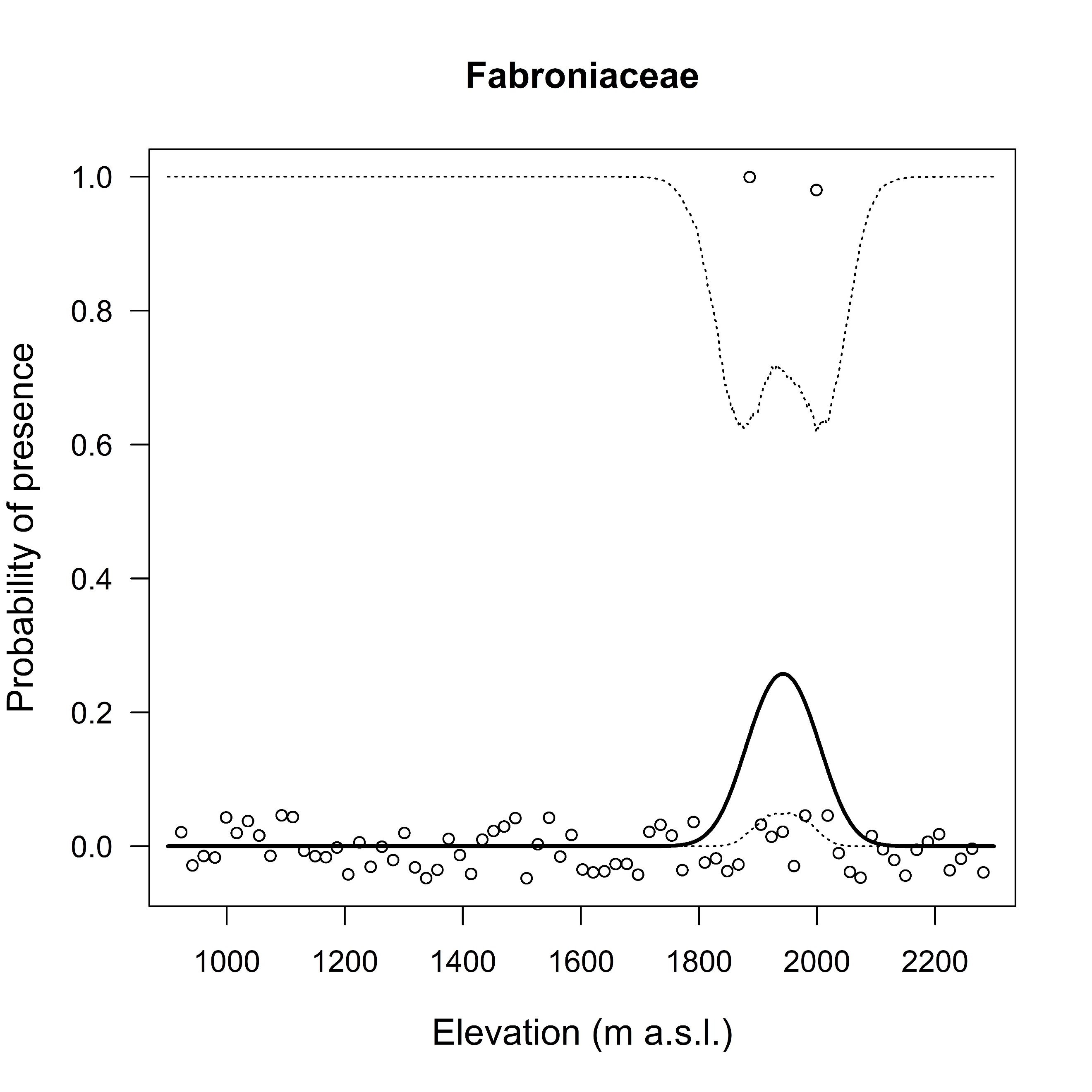 |
| 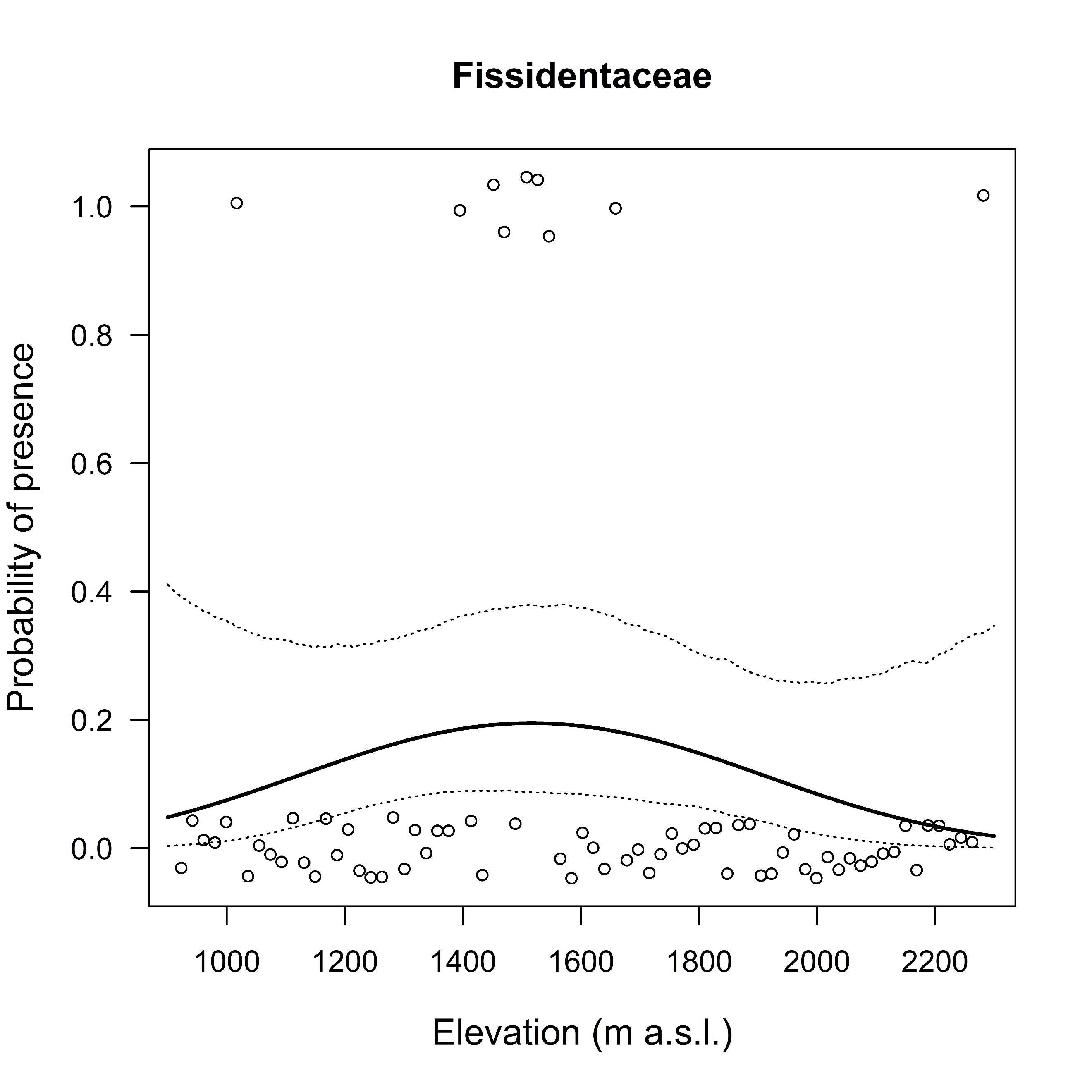 | 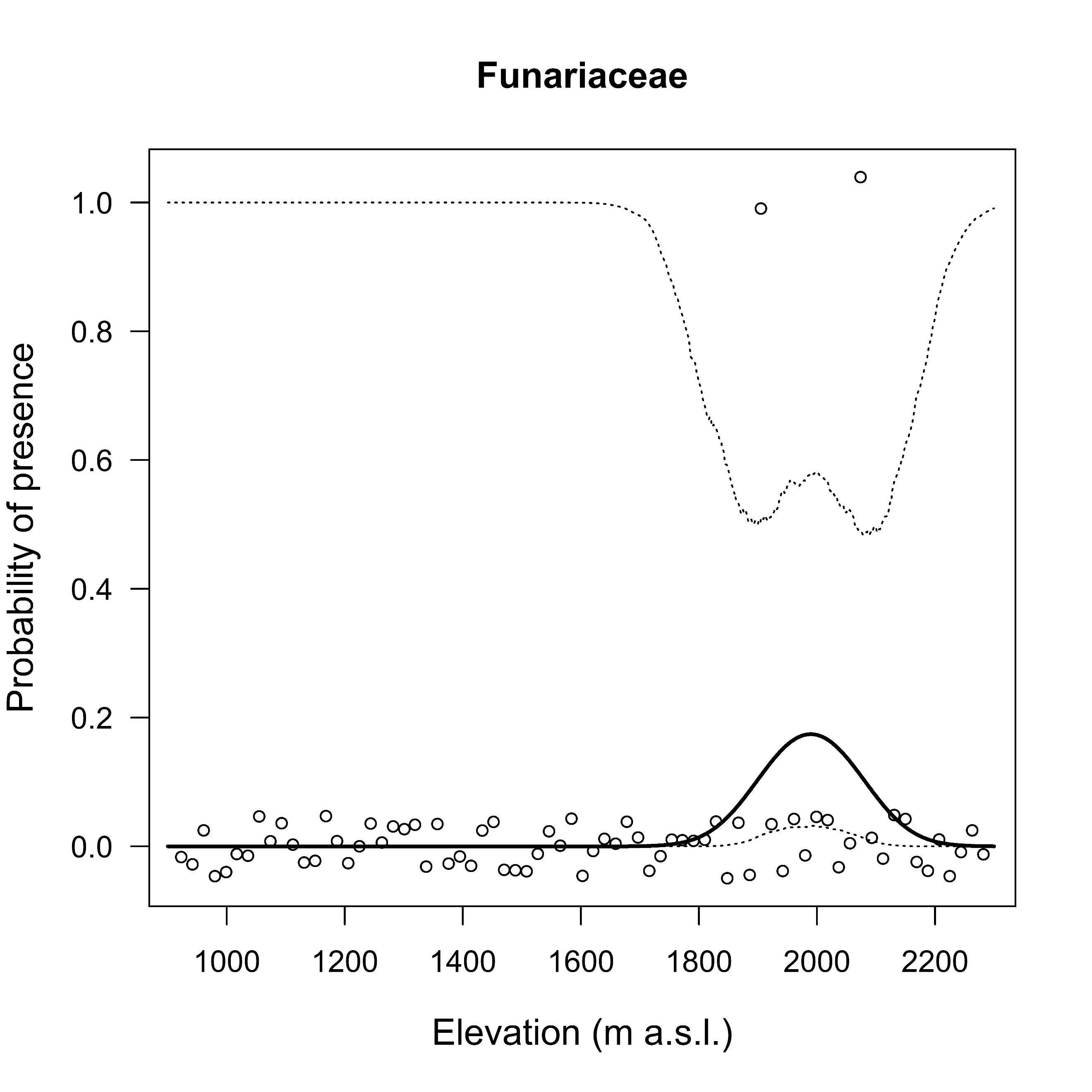 |
| 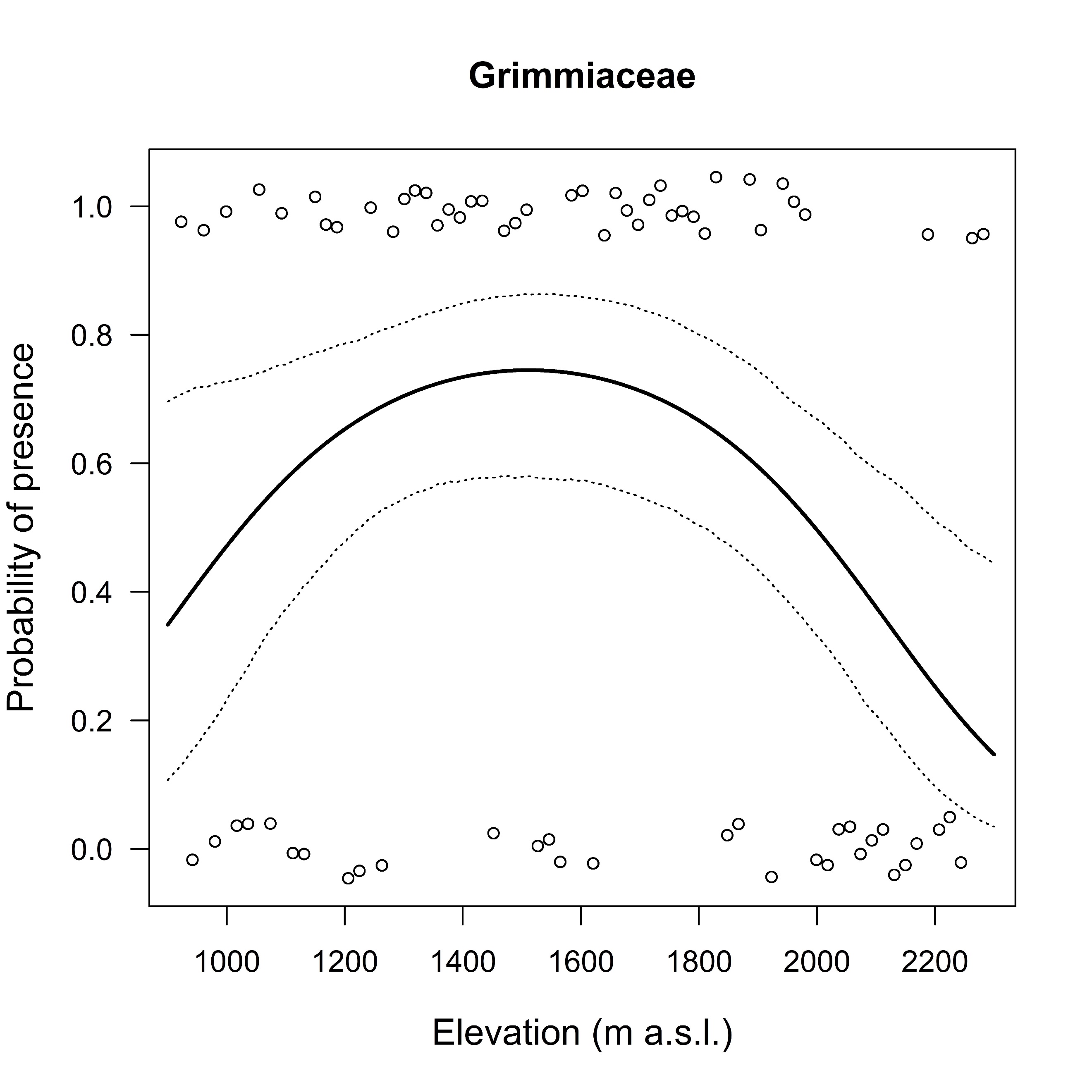 | 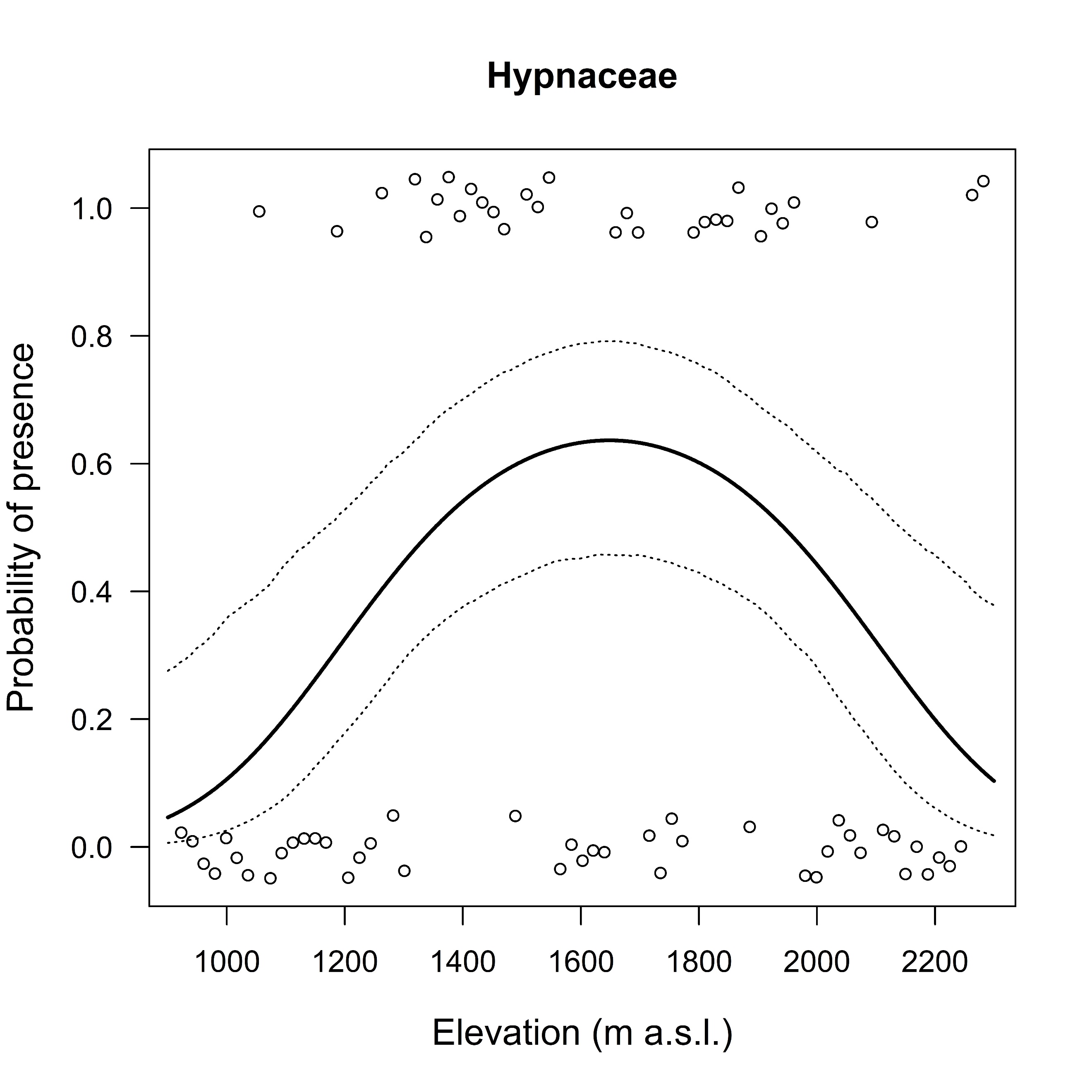 |
| 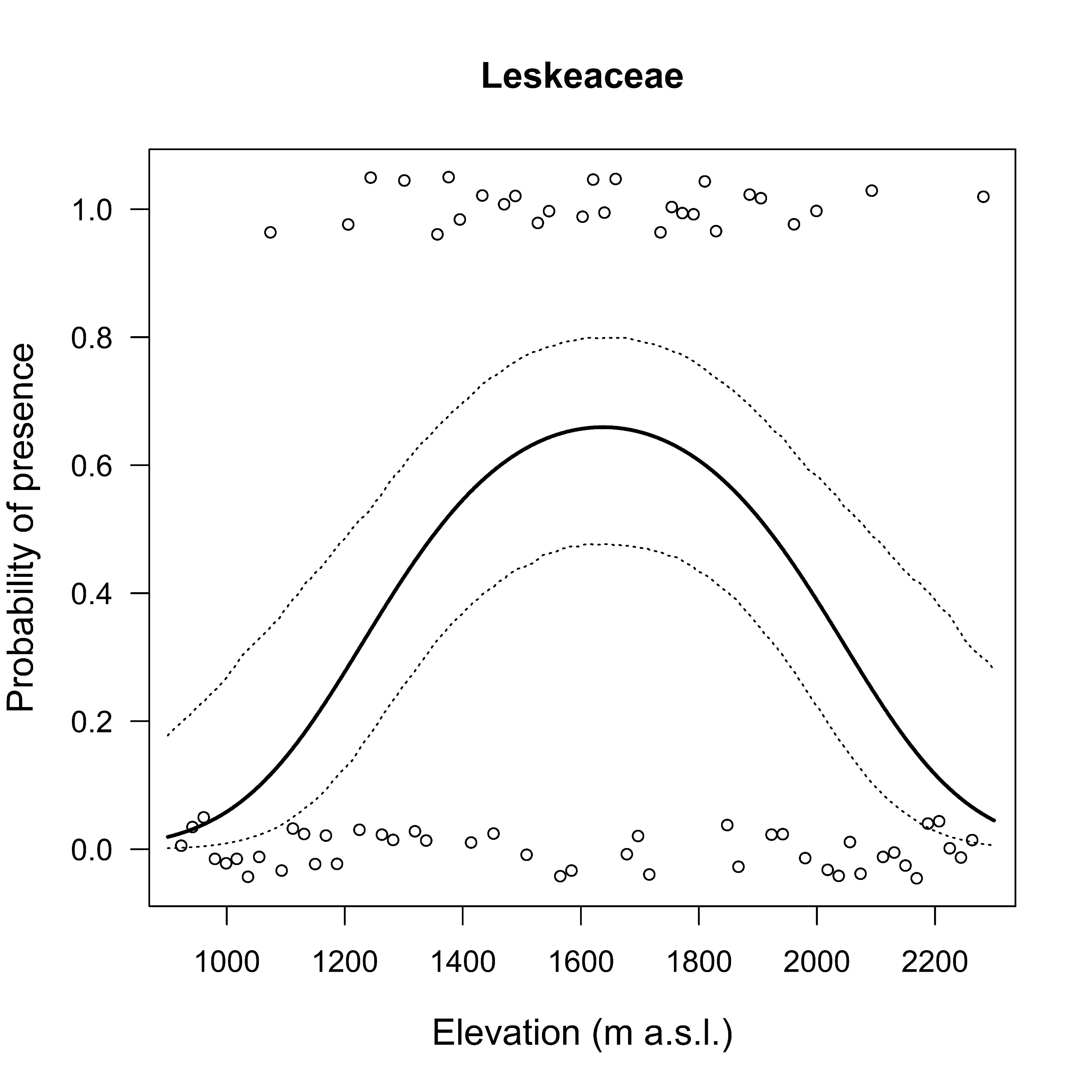 | 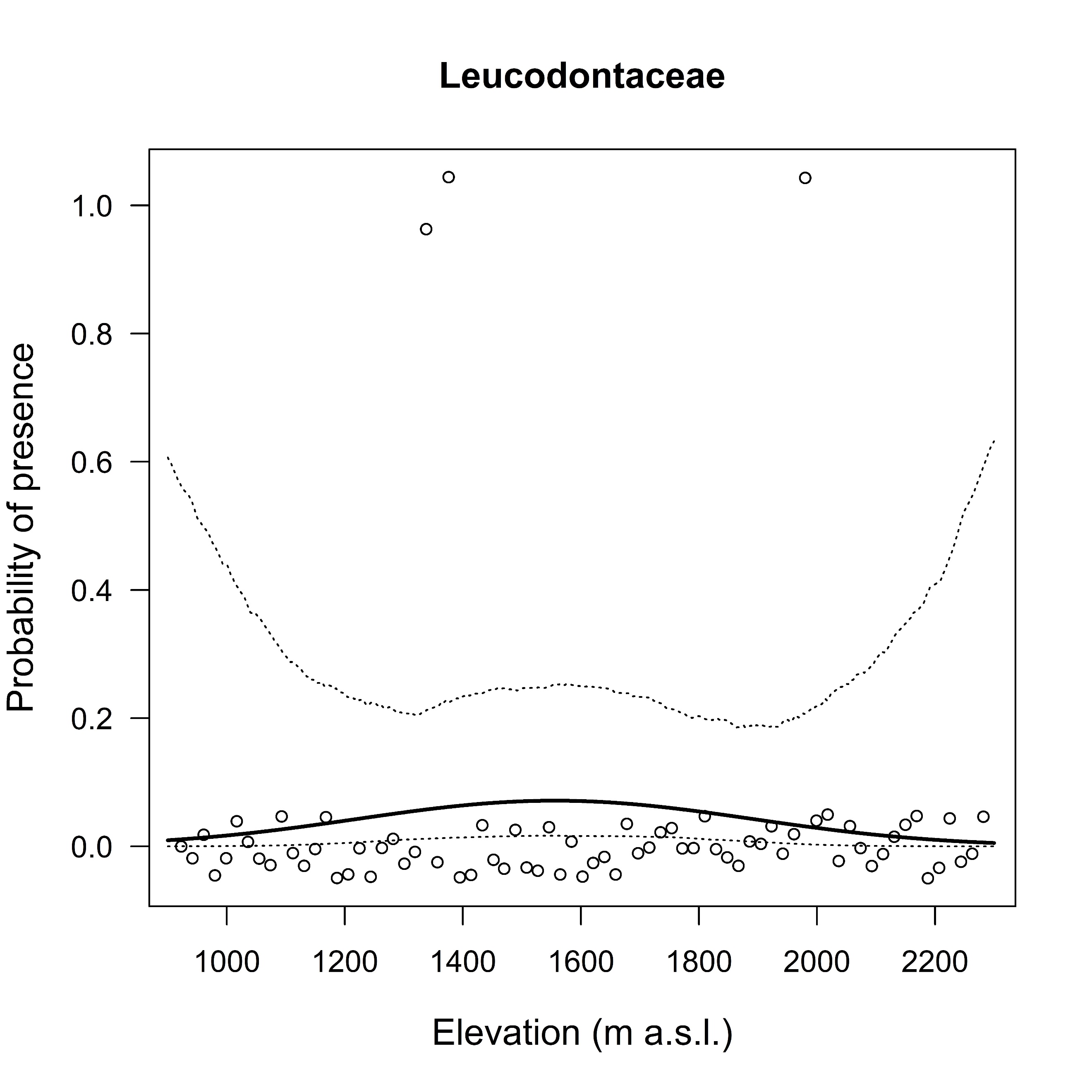 |
| 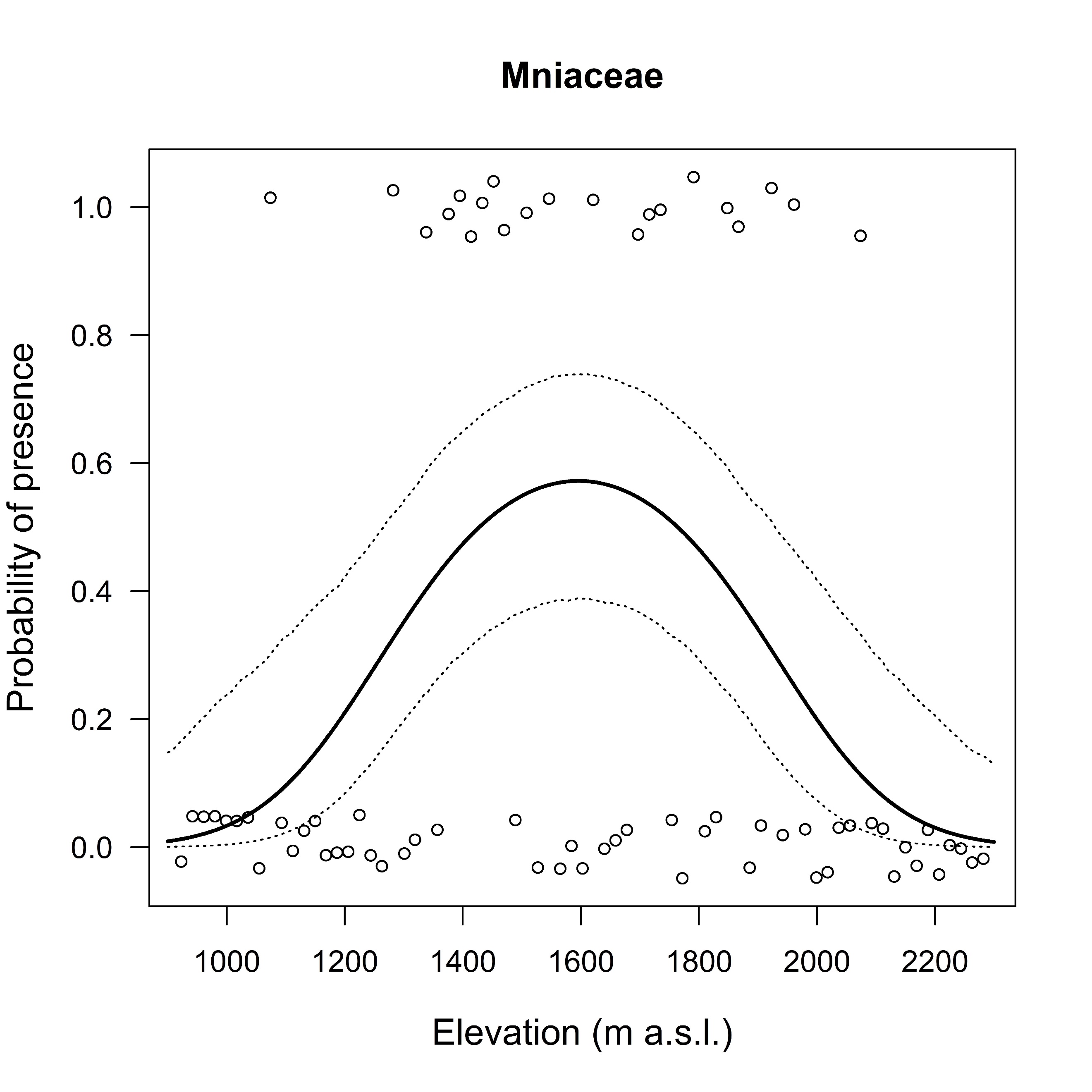 | 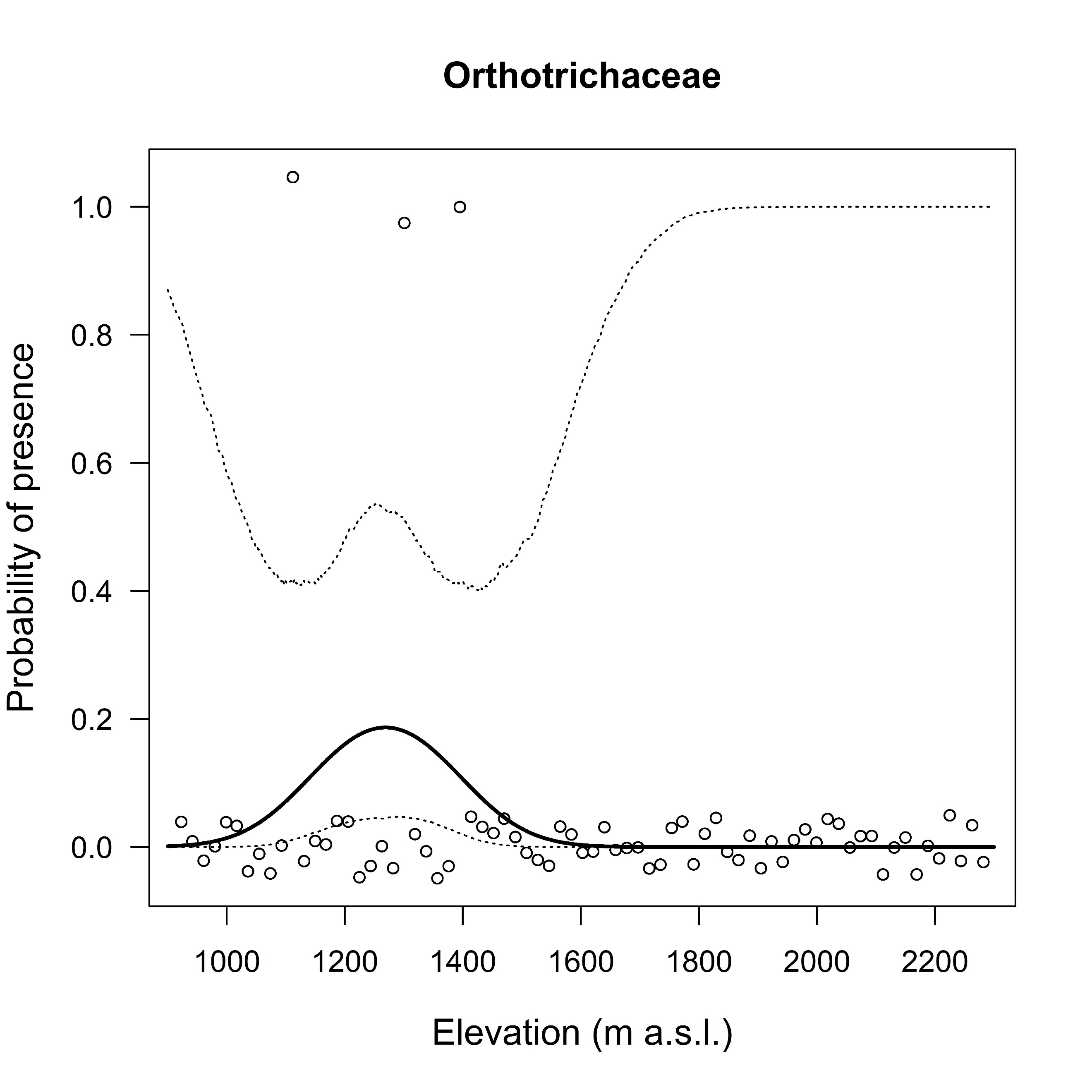 |
| 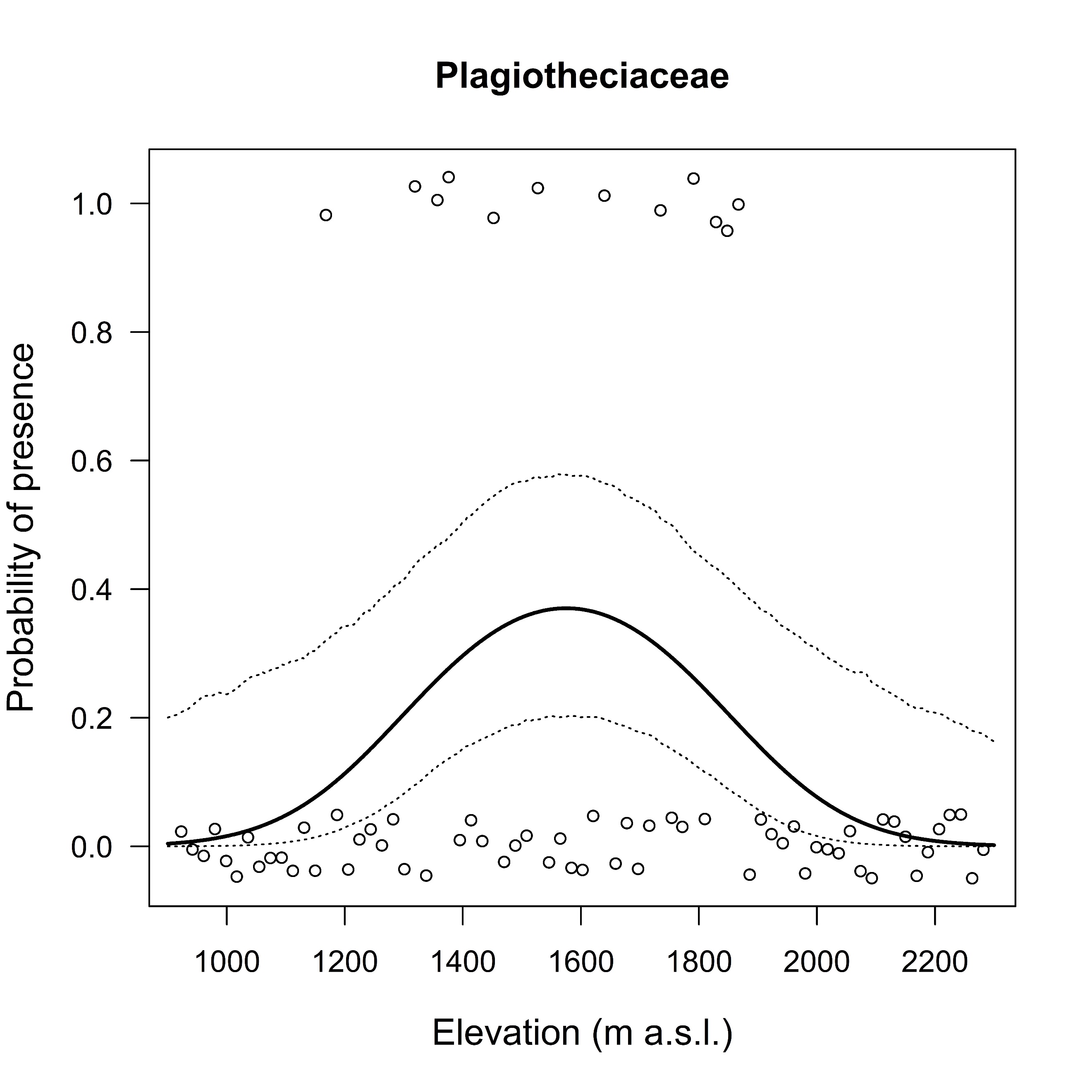 | 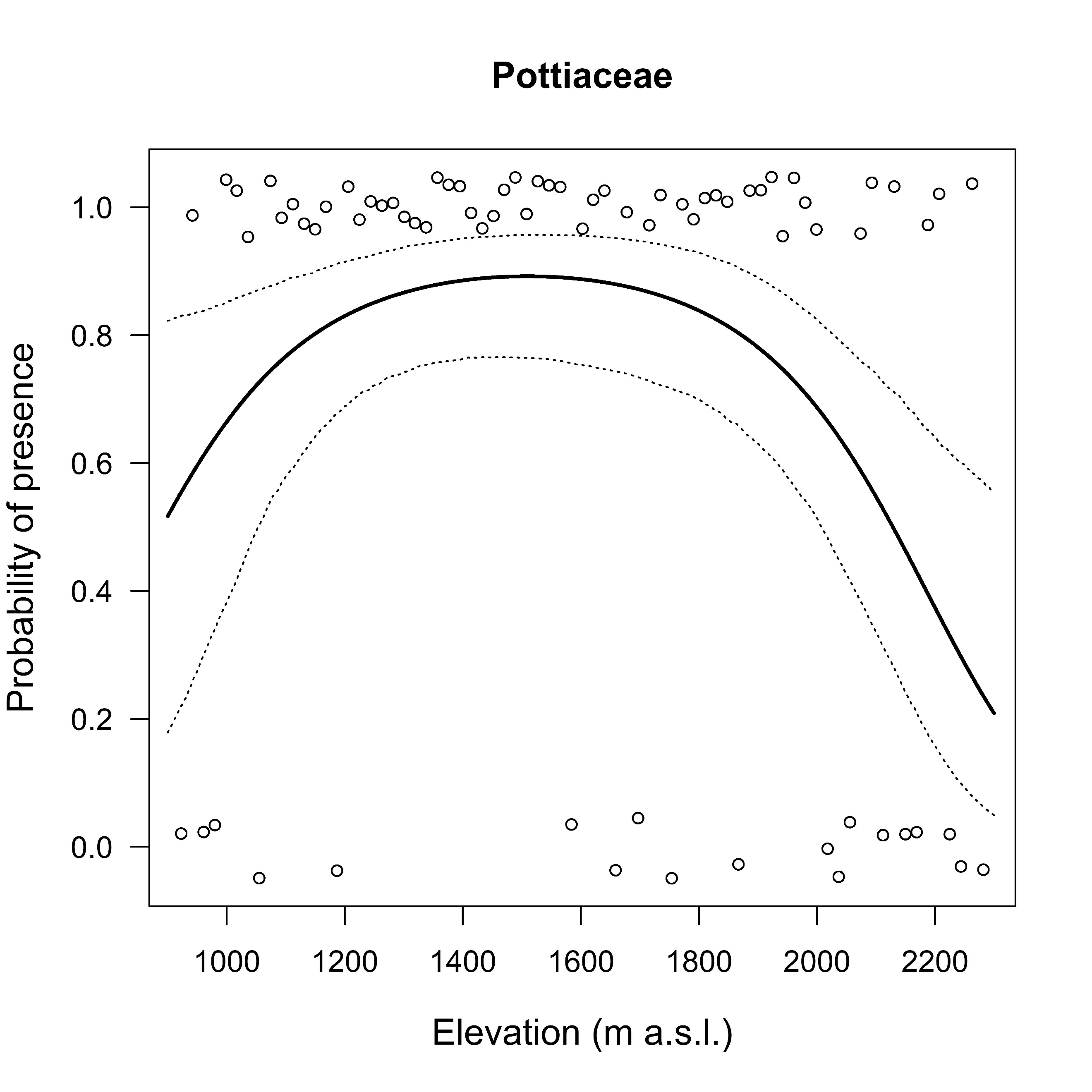 |
| 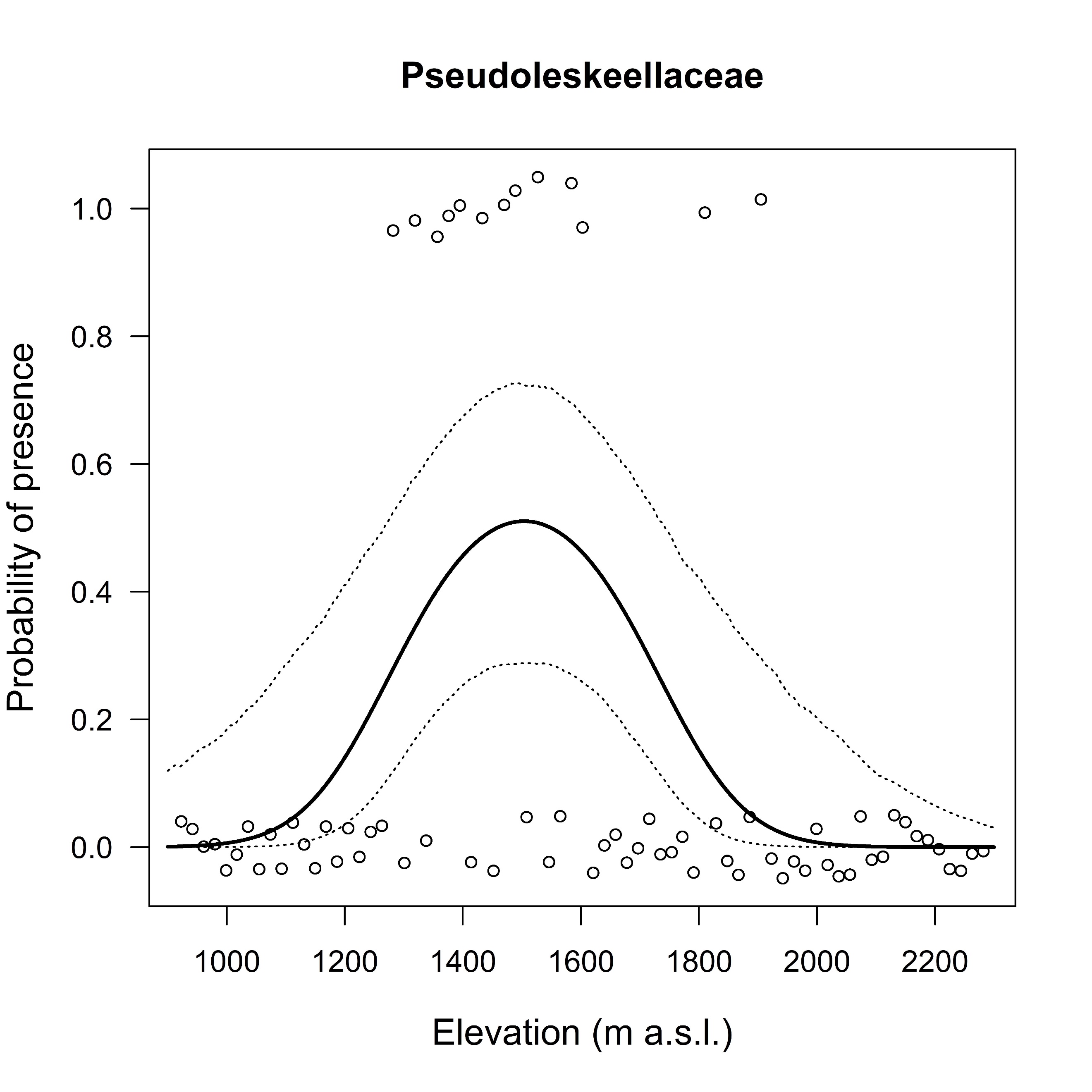 | 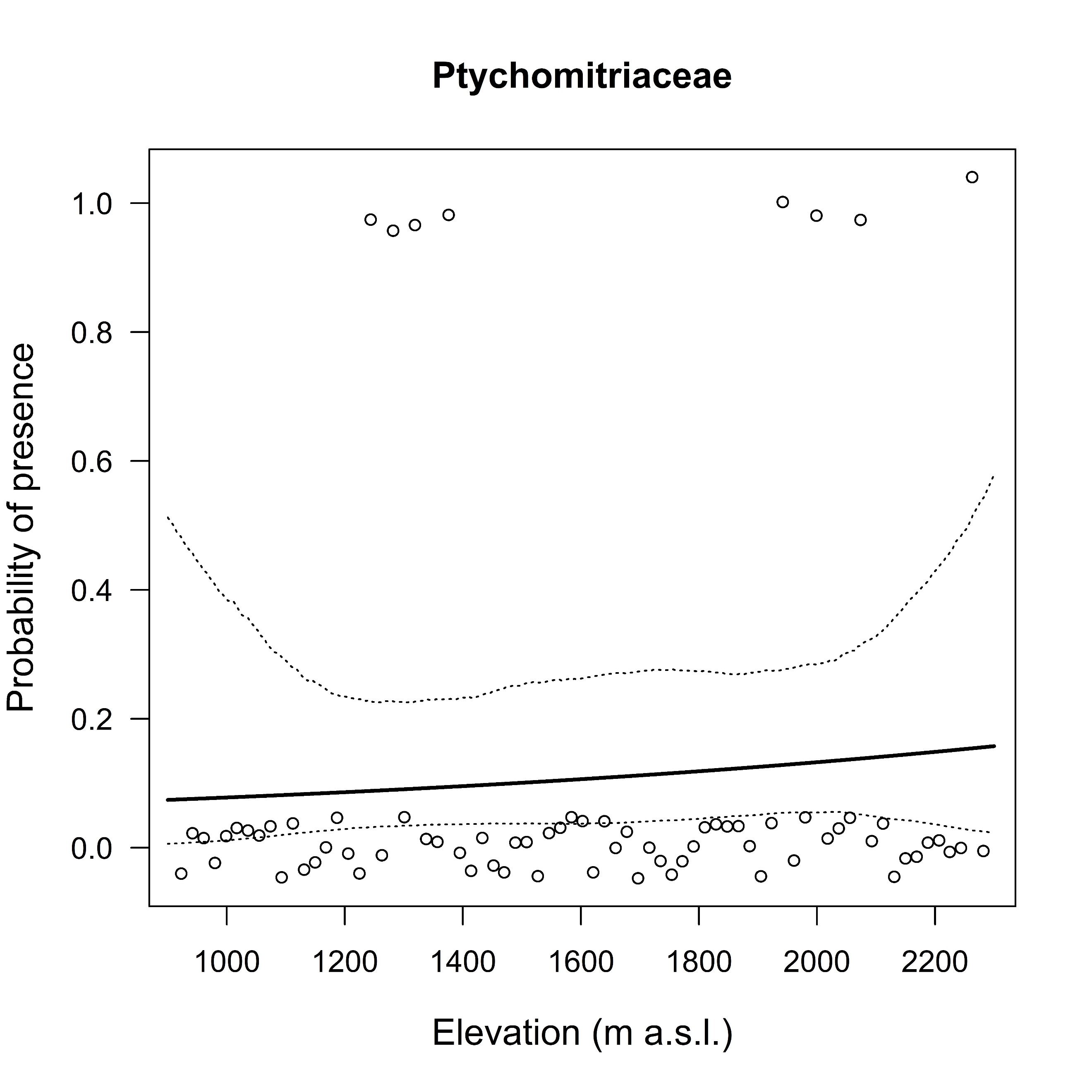 |
| 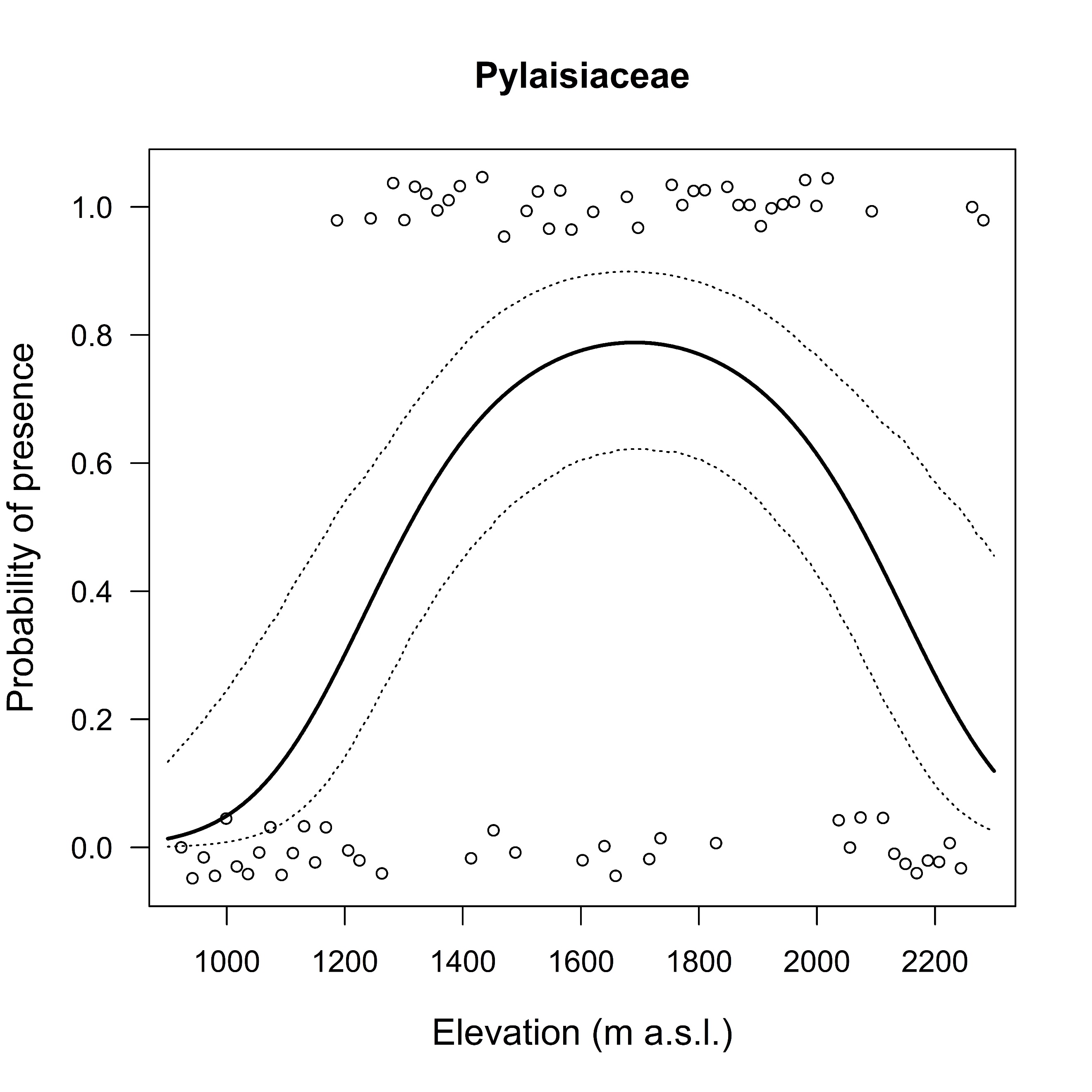 | 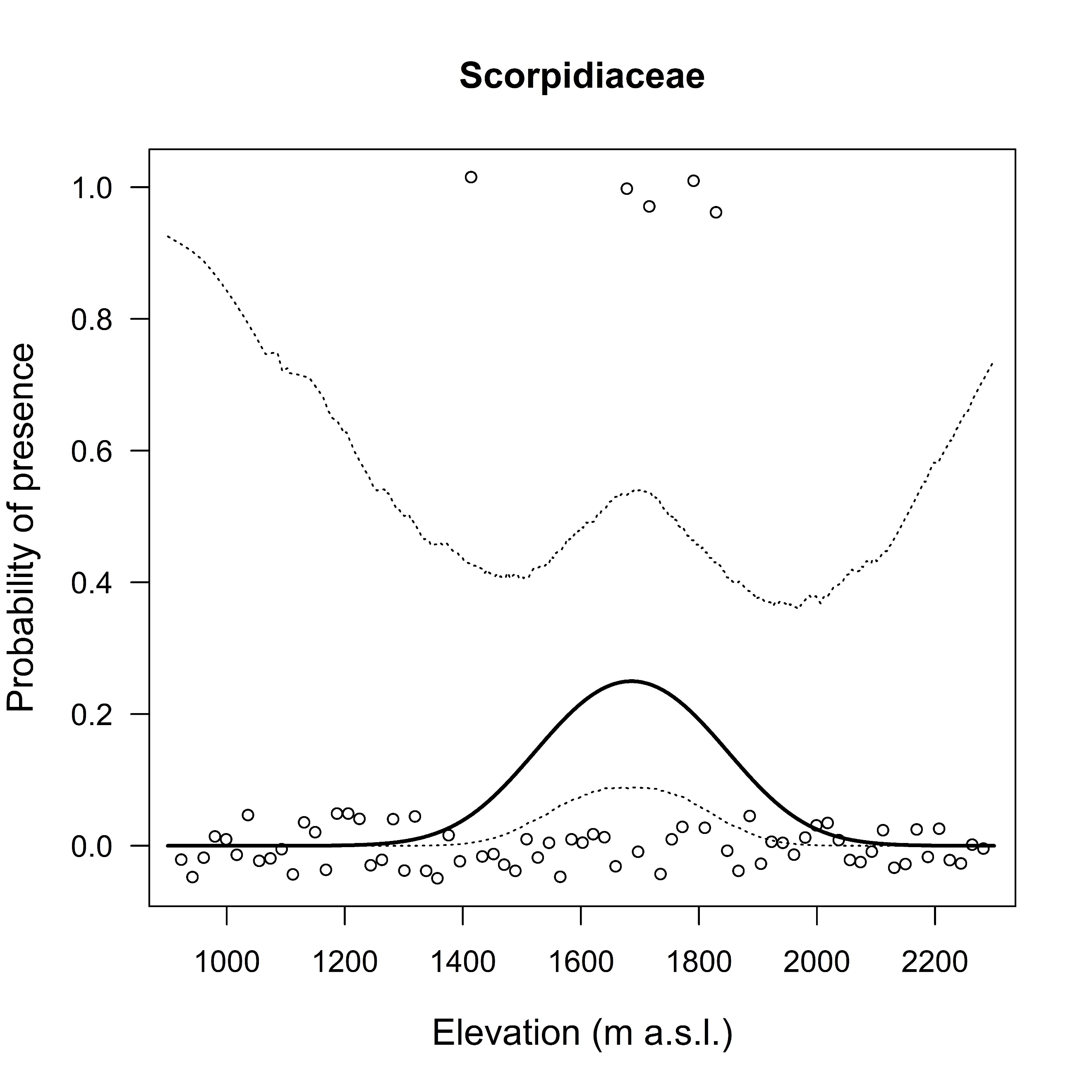 |
| 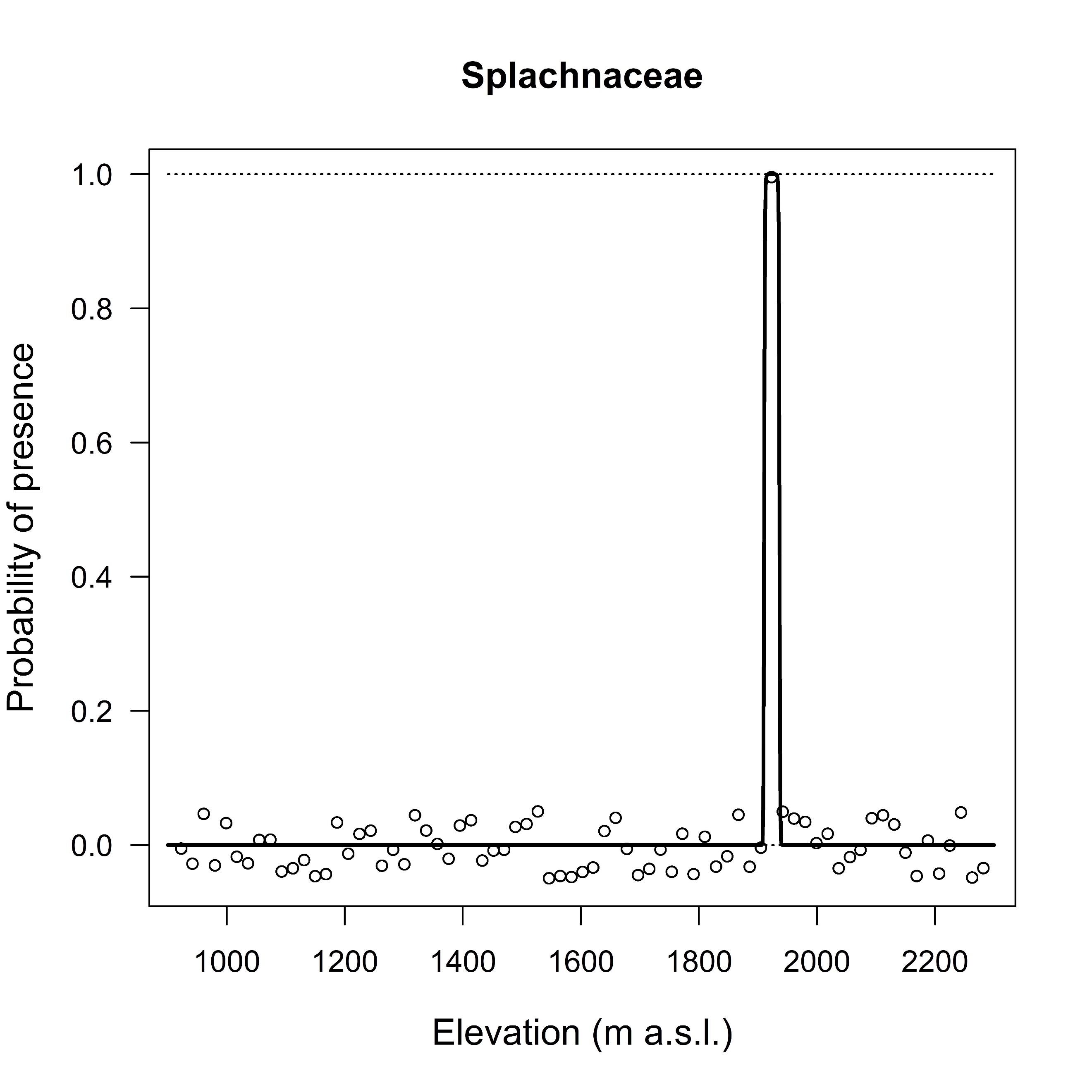 | 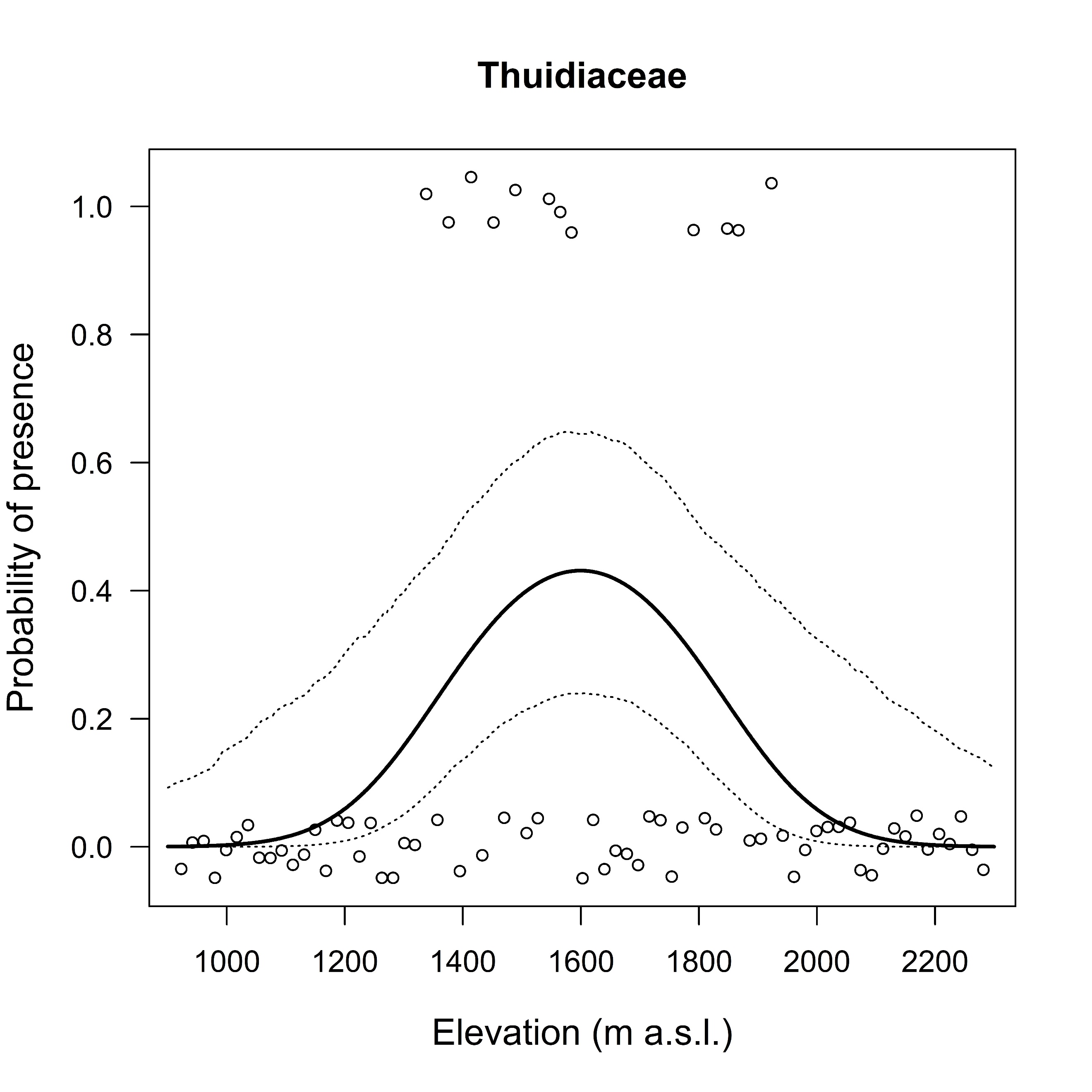 |
