# Supplementary material for: The mid‐domain effect and habitat complexity applied to elevational gradients: Moss species richness in a temperate semihumid monsoon climate mountain of China
Source: Ecol Evol. 2021 May 4;11(12):7448–60. doi: 10.1002/ece3.7576 (PMC8216932; doi:10.1002/ece3.7576)
Supplement: Supplementary file 4 — Table S3 [file ECE3-11-7448-s005.docx]

Table S3. Checklist of moss species identified in the 73 sampling sites. Asterisk indicates the species is endemic.

| Family | Genus | Species |
| --- | --- | --- |
| Encalyptaceae | *Encalypta* | *Encalypta ciliata* Hedw. |
| Funariaceae | *Funaria* | *Funaria hygromexrica* Hedw.* |
| Drummondiaceae | *Drummondia* | *Drummondia sinensis* Müll. Hal.* |
| Ptychomitriaceae | *Ptychomitrium* | *Ptychomitrium linearifolium* Reimers in Reimers & Sakurai |
|  |  | *Ptychomitrium sinense* (Mitt.) A. Jaeger |
| Grimmiaceae | *Grimmia* | *Grimmia laevigata* (Brid.) Brid. |
|  |  | *Grimmia longirostris* Hook. |
|  |  | *Grimmia montana* Bruch. & Schimp. |
|  |  | *Grimmia ovalis* (Hedw.) Lindb. |
|  |  | *Grimmia pilifera* P. Beauv. |
|  | *Schistidium* | *Schistidium apocarpum* (Hedw.) Bruch & Schimp. |
|  |  | *Schistidium liliputanum* (Müll. Hal.) Deguchi |
|  |  | *Schistidium rivulare* (Brid.) Podp. |
|  |  | *Schistidium subconfertum* (Broth.) Deguchi |
| Ditrichaceae | *Ceratodon* | *Ceratodon purpurens* (Hedw.) Brid. |
|  |  | *Ceratodon stenocarpus* Bruch & Schimp. |
|  | *Distichium* | *Distichium inclinatum* (Hedw.) Bruch & Schimp. |
|  | *Ditrichum* | *Ditrichum pusillum* (Hedw.) Hampe |
|  | *Pleuridium* | *Pleuridium subulatum* (Hedw.) Rabenh. |
| Fissidentaceae | *Fissidens* | *Fissidens bryoides* Hedw. |
|  |  | *Fissidens teysmannianus* Dozy & Molk. |
| Pottiaceae | *Anoectangium* | *Anoectangium aestivum* (Hedw.) Mitt. |
|  |  | *Anoectangium clarum* Mitt. |
|  |  | *Anoectangium stracheyanun* Mitt. |
|  |  | *Anoectangium thomsonii* Mitt. |
|  | *Barbula* | *Barbula gracilenta* Mitt. |
|  |  | *Barbula indica* (Hook.) Spreng. in Steud. |
|  |  | *Barbula unguiculata* Hedw. |
|  | *Brachymenium* | *Brachymenium acuminatum* Harv*.*in Hook. |
|  | *Bryoerythrophyllum* | *Bryoerythrophyllum recurvirostrum* (Hedw.) P. C. Chen |
|  |  | *Bryoerythrophyllum wallichii* (Mitt.) P. C. Chen |
|  | *Didymodon* | *Didymodon fallax* (Hedw.) Zander. |
|  |  | *Didymodon longicostatus* (X. J. Li) X. J. Li & Iwat. |
|  |  | *Didymodon rigidulus* Hedw. var. *rigidulus* |
|  |  | *Didymodon rigidulus* var. *icmadophilus* (Schimp. & C. Muell.) |
|  |  | *Didymodon rivicola* (Broth.) R. H. Zander |
|  |  | *Didymodon tectorus* (Müll. Hal.) Saito |
|  | *Gymnostomum* | *Gymnostomum calcareum* Nees & Hornsch. |
|  | *Hymenostylium* | *Hymenostylium recurvirostrum* (Hedw.) var. *recurvirostrum* |
|  |  | *Hymenostylium recurvirostrum* var. *cylindricum* (E. B. Bartram) R. H. Zander |
|  | *Hyophila* | *Hyophila sexschwanica* (Broth.) Hilp. & P. C. Chen |
|  |  | *Hyophila spathulata* (Harv.) A. Jaeg. |
|  | *Molendoa* | *Molendoa schliephackei* (Limpr.) R. H. Zander |
|  |  | *Molendoa sendtneriana* (Bruch & Schimp.) Limpr. |
|  | *Pseudosymblepharis* | *Pseudosymblepharis angustata* (Mitt.) Hilp. |
|  | *Syntrichia* | *Syntrichia fragilis* (Taylor) Ochyra |
|  |  | *Syntrichia gemmascens* (P. C. Chen) R. H. Zander |
|  |  | *Syntrichia norvegica* F. Weber |
|  |  | *Syntrichia sinnesis* (Müll. Hal.) Ochyra |
|  | *Timmiella* | *Timmiella anomala* (Bruch & Schimp) Limpr. |
|  |  | *Timmiella diminuta* (Müll. Hal.) P. C. Chen |
|  | *Tortula* | *Tortula leucostoma* (R. Br. bis) Hook. & Grev. |
|  |  | *Tortula mucronifolia* Schwägr. |
|  |  | *Tortula planifolia* X. J. Li |
|  |  | *Tortula truncata* (Hedw.) Mitt. |
|  | *Trichostomum* | *Trichostomum brachydontium* Bruch |
|  |  | *Trichostomum crispulum* Bruch in F. A. Müll. |
|  |  | *Trichostomum hattorianum* B. C. Tan & Z. Iwats. |
|  | *Weissia* | *Weissia edentula* Mitt. |
|  |  | *Weissia exserta* (Broth.) P. C. Chen |
|  |  | *Weissia longifolia* Mitt. |
|  |  | *Weissia newcomeri* (E. B. Bartram) Saito |
| Bartramiaceae | *Dicranella* | *Dicranella heteromalla* (Hedw.) Schimp. |
| Splachnaceae | *Tayloria* | *Tayloria indica* Mitt.* |
| Bryaceae | *Anomobryum* | *Anomobryum gemmigerum* Broth. |
|  |  | *Anomobryum julaceum* (Gärtn. Meyer & Scherb.) Schimp. |
|  | *Brachymenium* | *Brachymenium capitulatum* (Mitt.) Kindb. |
|  |  | *Brachymenium exile* (Dozy. & Molk.) Bosch & Lac. |
|  |  | *Brachymenium sinensse* Cardot & Thér. |
|  | *Bryum* | *Bryum alpinum* Huds. & With. |
|  |  | *Bryum argenteum* Hedw. |
|  |  | *Bryum billarderi* Schwägr. |
|  |  | *Bryum bornholmense* Winkelm. & Ruthe. |
|  |  | *Bryum caespiticium* Hedw. |
|  |  | *Bryum capillare* Hedw. |
|  |  | *Bryum coronatum* Schwägr |
|  |  | *Bryum lonchocaulon* Müll. Hal. |
|  |  | *Bryum pallescens* Schleich. & Schwägr. |
|  |  | *Bryum paradoxum* Schwaegr. |
|  |  | *Bryum pseudotriquexrum* (Hedw.) Gaertn. |
|  |  | *Bryum tuberosum* Mohamed & Damanhuri |
|  |  | *Bryum uliginosum* (Brid.) Bruch & Schimp. |
|  | *Plagiobryum* | *Plagiobryum giraldii* (Müll. Hal.) Paris |
|  | *Rhodobryum* | *Rhodobryum giganteum* (Schwägr.) Paris |
| Mniaceae | *Mnium* | *Mnium marginatum* (With.) P. Beauv. |
|  |  | *Mnium spinosum* (Voit) Schwägr. |
|  |  | *Mnium spinulosum* Bruch & Schimp. |
|  | *Plagiomnium* | *Plagiomnium acutum* (Lindb.) T. J. Kop. |
|  |  | *Plagiomnium cuspidatum* (Hedw.) T. J. Kop. |
|  |  | *Plagiomnium drummondii* (Bruch & Schimp.) T. J. Kop. |
|  |  | *Plagiomnium integrum* (Bosch & Sande Lac.) T. J. Kop. |
|  |  | *Plagiomnium succulentum* (Mitt.) T. J. Kop. |
|  | *Trachycystis* | *Trachycystis ussuriensis* (Maack & Regel) T. J. Kop. |
| Orthotrichaceae | *Orthotrichum* | *Orthotrichum sordidum* Sull. & Lesq. |
|  |  | *Orthotrichum anomalum* Hedw. |
| Plagiotheciaceae | *Herzogiella* | *Herzogiella perrobusta* (Broth. & Cardot) Z. Iwats |
|  |  | *Herzogiella seligeri* (Brid.) Z. Iwats |
|  |  | *Herzogiella striatella* (Brid.) Z. Iwats |
|  | *Plagiothecium* | *Plagiothecium cavifolium* var. *cavifolium* |
|  |  | *Plagiothecium cavifolium* (Brid.) Iwats. var. *fallax* (Card. & Thér) Z. Iwats |
|  |  | *Plagiothecium handelii* Broth. |
|  | *Pseudotaxiphyllum* | *Pseudotaxiphyllum densum* (Cardot) Z. Iwats. |
| Fabroniaceae | *Fabronia* | *Fabronia ciliaris* (Brid.) Brid. |
| Amblystegiaceae | *Amblystegium* | *Amblystegium serpens* (Hedw.) Bruch & Schimp. |
|  |  | *Amblystegium varium* (Hedw.) Lindb. |
|  | *Campyliadelphs* | *Campyliadelphs polygamum* (Bruch & Schimp.) Kanda |
|  |  | *Campyliadelphs stellatus* (Hedw.) Kanda |
|  | *Cratoneuron* | *Cratoneuron filinum* (Hedw.) Spruce |
| Scorpidiaceae | *Sanionia* | *Sanionia uncinata* (Hedw.) Loeske* |
| Leskeaceae | *Clapodium* | *Clapodium leptopteris* (Müll. Hal.) P. C. Wu & M. Z. Wang |
|  | *Leptoterigynandrum* | *Leptoterigynandrum austro-alpinum* Müll. Hal. |
|  |  | *Leptoterigynandrum subintegrum* (Mitt.) Broth. |
|  | *Leskeella* | *Leskeella nervosa* (Brid.) Loeske |
|  |  | *Lindbergia brachyptera* (Mitt.) Kindb. |
|  |  | *Lindbergia sinensis* (Müll. Hal.) Broth. |
| Pseudoleskeellaceae | *Pseudoleskeella* | *Pseudoleskeella tectorrum* (Brid.) Kindb. |
| Thuidiaceae | *Bryonoguchia* | *Bryonoguchia molkenboeri* (Sande Lac.) Z. Iwats. & Inoue |
|  | *Haplocladium* | *Haplocladium angustifolium* (Hamp. & Müll. Hal.) Broth. |
|  |  | *Haplocladium microphyllum* (Hedw.) Broth. |
|  | *Rauiella* | *Rauiella fujisana* (Paris) Reimers |
|  | *Thuidium* | *Thuidium assimile* (Mitt.) A. Jaeger |
|  |  | *Thuidium cymbifom* (Dozy & Molk.) Dozy & Molk. |
|  |  | *Thuidium delicatulum* (Hedw.) Schimp. |
|  |  | *Thuidium kanedae* Sakurai |
|  |  | *Thuidium plumulosum* (Dozy & Molk.) Dozy & Molk. |
| Brachytheciaceae | *Brachythecium* | *Brachythecium buchananii* (Hook.) A. Jaeger |
|  |  | *Brachythecium campylothallum* Müll. Hal. |
|  |  | *Brachythecium erythrorrhizon* Bruch & Schimp. |
|  |  | *Brachythecium garovaglioides* Müll. Hal. |
|  |  | *Brachythecium glareosum* (Spruce) Bruch & Schimp. |
|  |  | *Brachythecium glauculum* C. Muell. |
|  |  | *Brachythecium homocladum* Müll. Hal. |
|  |  | *Brachythecium kuroishicum* Besch. |
|  |  | *Brachythecium noguchii* Takaki. |
|  |  | *Brachythecium perscabrum* Broth. |
|  |  | *Brachythecium piligerum* Cardot |
|  |  | *Brachythecium pinnirameum* Müll. Hal. |
|  |  | *Brachythecium pulchellum* Broth. & Paris |
|  |  | *Brachythecium rivulare* Bruch & Schimp. |
|  |  | *Brachythecium rotaeanum* De Not. |
|  |  | *Brachythecium rutabulum* (Hedw.) Bruch & Schimp. |
|  |  | *Brachythecium salebrosum* (F. Weber & D. Mohr) Bruch & Schimp. |
|  |  | *Brachythecium yunnanense* Herzog |
|  | *Bryhnia* | *Bryhnia novae-angliae* (Sull. & Lesq.) Grout |
|  | *Eurhynchium* | *Eurhynchium angustirete* (Broth.) T. J. Kop. |
|  | *Homalothecium* | *Homalothecium laevisexum* Sande Lac. |
|  | *Myuroclada* | *Myuroclada maximowiczii* (G. G. Borshch.) Steere & W. B. Schofield |
|  | *Okamuraea* | *Okamuraea brachydictyon* (Cardot) Nog. |
|  | *Oxyrrhynchium* | *Oxyrrhynchium laxirete* (Broth.) Broth. |
|  | *Palamocladium* | *Palamocladium leskeoides* (Hook.) E. Britton |
|  | *Rhynchostegiella* | *Rhynchostegiella laeviseta* Broth. |
|  | *Rhynchostegium* | *Rhynchostegium fauriei* Cardot |
|  |  | *Rhynchostegium ovalifolium* S. Okamura |
|  |  | *Rhynchostegium pallidifolium* (Mitt.) A. Jaeger |
|  | *Sciuro-hypnum* | *Sciuro-hypnum brotheri* (Paris.) Ignatov & Huttunen |
|  |  | *Sciuro-hypnum curtum* (Lindb.) Ignatov & Huttunen |
|  |  | *Sciuro-hypnum glaciale* (Schimp.) Ignatov & Huttunen |
|  |  | *Sciuro-hypnum plumosum* (Hedw.) Ignatov & Huttunen |
|  |  | *Sciuro-hypnum reflexum* (Stark.) Ignatov & Huttunen |
|  |  | *Sciuro-hypnum starkii* (Brid.) Ignatov & Huttunen |
| Hypnaceae | *Ectropothecium* | *Ectropothecium obtusulum* (Cardot) Z. Iwats. |
|  | *Hypnum* | *Hypnum cupressiforme* Hedw. |
|  |  | *Hypnum fauriei* Cardot |
|  |  | *Hypnum leptothallum* (Müll. Hal.) Paris |
|  |  | *Hypnum macrogynum* Besch. |
|  |  | *Hypnum revolutum* (Mitt.) Lindb. |
|  |  | *Hypnum subimponens* Lesq. subsp. *ulophyllum* (Müll. Hal.) Ando |
|  |  | *Hypnum vaucheri* Lesq. |
|  | *Taxiphyllum* | *Taxiphyllum cuspidifolium* (Cardot) Z. Iwats. |
|  |  | *Taxiphyllum taxirameum* (Mitt.) M. Fleisch. |
| Pylaisiaceae | *Homomallium* | *Homomallium connexum* (Card.) Broth. |
|  |  | *Homomallium plagiangium* (Müll. Hal.) Broth. |
|  | *Pylaisiella* | *Pylaisiella brotheri* Besch. |
|  |  | *Pylaisiella polyantha* (Hedw.) Bruch & Schimp. |
| Entodontaceae | *Entodon* | *Entodon challengerii* (Paris) Cardot |
|  |  | *Entodon cladorrhizans* (Hedw.) Müll. Hal. |
|  |  | *Entodon concinnus* (De Not.) Paris |
|  |  | *Entodon giraldii* Müll. Hal. |
|  |  | *Entodon luridus* (Griff.) A. Jaeger |
|  |  | *Entodon morrisonensis* Nog. |
|  |  | *Entodon obtusatus* Broth. |
|  |  | *Entodon plicatus* Müll. Hal. |
|  |  | *Entodon pylaisioides* R. L. Hu. & Y. F. Wang |
|  |  | *Entodon schleicheri* Müll. Hal. |
|  |  | *Entodon smaragdinus* Paris & Broth. |
|  |  | *Entodon sullivantii* (Müll. Hal.) Lindb. |
| Leucodontaceae | *Leucodon* | *Leucodon pendulus* Lindb. |
|  |  | *Leucodon sciuroides* (Hedw.) Schwägr. |
| Anomodontaceae | *Anomodon* | *Anomodon minor* (Hedw.) Lindb. |
|  |  | *Anomodon viticulosus* (Hedw.) Hook. & Taylor |
|  | *Herpetineuron* | *Herpetineuron toccoae* (Sull. & Lesq.) Cardot |
