# Supplementary material for: The mid‐domain effect and habitat complexity applied to elevational gradients: Moss species richness in a temperate semihumid monsoon climate mountain of China
Source: Ecol Evol. 2021 May 4;11(12):7448–60. doi: 10.1002/ece3.7576 (PMC8216932; doi:10.1002/ece3.7576)
Supplement: Supplementary file 5 — Supplementary Material [file ECE3-11-7448-s002.docx]

**SUPPORTING INFORMATION**

Additional Supporting Information may be found online in the supporting information tab for this article:

**Table S1.** Occurrence data for moss species along elevational gradient. In the body of the table, 1 indicates presence, 0 indicates that the species was not detected at that sampling site.

**Table S2.** Environmental variables and richness data along elevational gradient.

**Table S3.** Checklist of moss species identified in the 73 sampling sites.

**Figure S1.** Moss presence/absence data versus elevation with regression line (black line) and 95% credible interval (dotted lines) for 26 families listed in alphabetical order. Open circles represent observed presence (1) or absence (0) that jittered in the vertical direction.
